# Tissue-specific transcriptomics and proteomics of a filarial nematode and its *Wolbachia* endosymbiont

**DOI:** 10.1186/s12864-015-2083-2

**Published:** 2015-11-11

**Authors:** Ashley N. Luck, Kathryn G. Anderson, Colleen M. McClung, Nathan C. VerBerkmoes, Jeremy M. Foster, Michelle L. Michalski, Barton E. Slatko

**Affiliations:** Genome Biology Division, New England Biolabs, Inc., 240 County Road, Ipswich, MA 01938 USA; Department of Biology and Microbiology, University of Wisconsin Oshkosh, Oshkosh, WI 54901 USA; Chemical Biology Division, New England Biolabs, Inc., 240 County Road, Ipswich, MA 01938 USA

**Keywords:** Nematode, Filaria, Transcriptomics, Proteomics, Endosymbiosis, *Wolbachia*

## Abstract

**Background:**

Filarial nematodes cause debilitating human diseases. While treatable, recent evidence suggests drug resistance is developing, necessitating the development of novel targets and new treatment options. Although transcriptomic and proteomic studies around the nematode life cycle have greatly enhanced our knowledge, whole organism approaches have not provided spatial resolution of gene expression, which can be gained by examining individual tissues. Generally, due to their small size, tissue dissection of human-infecting filarial nematodes remains extremely challenging. However, canine heartworm disease is caused by a closely related and much larger filarial nematode, *Dirofilaria immitis*. As with many other filarial nematodes, *D. immitis* contains *Wolbachia,* an obligate bacterial endosymbiont present in the hypodermis and developing oocytes within the uterus. Here, we describe the first concurrent tissue-specific transcriptomic and proteomic profiling of a filarial nematode (*D. immitis)* and its *Wolbachia* (*w*Di) in order to better understand tissue functions and identify tissue-specific antigens that may be used for the development of new diagnostic and therapeutic tools.

**Methods:**

Adult *D. immitis* worms were dissected into female body wall (FBW), female uterus (FU), female intestine (FI), female head (FH), male body wall (MBW), male testis (MT), male intestine (MI), male head (MH) and 10.1186/s12864-015-2083-2 male spicule (MS) and used to prepare transcriptomic and proteomic libraries.

**Results:**

Transcriptomic and proteomic analysis of several *D. immitis* tissues identified many biological functions enriched within certain tissues. Hierarchical clustering of the *D. immitis* tissue transcriptomes, along with the recently published whole-worm adult male and female *D. immitis* transcriptomes, revealed that the whole-worm transcriptome is typically dominated by transcripts originating from reproductive tissue. The uterus appeared to have the most variable transcriptome, possibly due to age. Although many functions are shared between the reproductive tissues, the most significant differences in gene expression were observed between the uterus and testis. Interestingly, *w*Di gene expression in the male and female body wall is fairly similar, yet slightly different to that of *Wolbachia* gene expression in the uterus. Proteomic methods verified 32 % of the predicted *D. immitis* proteome, including over 700 hypothetical proteins of *D. immitis*. Of note, hypothetical proteins were among some of the most abundant *Wolbachia* proteins identified, which may fulfill some important yet still uncharacterized biological function.

**Conclusions:**

The spatial resolution gained from this parallel transcriptomic and proteomic analysis adds to our understanding of filarial biology and serves as a resource with which to develop future therapeutic strategies against filarial nematodes and their *Wolbachia* endosymbionts.

**Electronic supplementary material:**

The online version of this article (doi:10.1186/s12864-015-2083-2) contains supplementary material, which is available to authorized users.

## Background

Afflicting nearly 150 million people, filarial nematodes responsible for lymphatic filariasis (*Wuchereria bancrofti*, *Brugia malayi* and *B. timori)* and onchocerciasis (*Onchocerca volvulus*) are a worldwide source of disablement and morbidity [1]. Although current microfilaricidal treatments (which kill the larval stages) disrupt transmission of these insect vector-borne diseases [2], treatment is contraindicated in certain regions due to adverse drug reactions in the presence of another filarial nematode, *Loa loa*. Additionally, because recent evidence suggests drug resistance in filarial nematodes is developing [3, 4], the identification of novel vaccine targets and the development of new treatment options are necessary.

Many filarial nematode species (including *W. bancrofti*, *B. malayi*, *B. timori* and *O. volvulus*) contain *Wolbachia*, an obligate intracellular endosymbiotic bacterium found in the lateral cords of all adult worms as well as within the oocytes and developing embryos in the female reproductive tract [5–7]. Depletion of *Wolbachia* through tetracycline antibiotic treatment leads to decreased fertility and eventually death of adult worms [8–10]. Genome sequencing of filarial nematodes and their *Wolbachia* endobacteria has identified a number of critical metabolites implicated in the host-endosymbiont interaction [11–13]. For example, most filarial nematodes lack genes necessary for the synthesis of critical cofactors such as heme and riboflavin, as well as the *de novo* synthesis of purines and pyrimidines while these pathways are complete and likely functional within their corresponding *Wolbachia* genomes [11, 14]. On the other hand, *Wolbachia* only partially maintains biosynthetic pathways for the synthesis of Coenzyme A, NAD, biotin, lipoic acid, ubiquinone and pyridoxal phosphate and thus, these cofactors may be supplied to the bacterium by the nematode host [11, 14].

While increasingly more filarial nematode genome sequences (and their respective *Wolbachia* counterparts) are becoming readily available, other ‘omics’ approaches (transcriptomics/proteomics) are needed to provide more detailed functional information. Previous transcriptomics (RNA-seq) studies have analyzed gene expression throughout the filarial nematode life cycle [15, 16]. Nevertheless, these whole organism approaches do not provide spatial resolution of gene expression, which can be gained by tissue specific gene expression profiling. Given the relatively small size of many filarial nematodes (*B. malayi* adults typically range from 13 to 55 mm in length), tissue dissection is extremely challenging. Hence, information on tissue-specific gene expression in nematodes has been limited to the widely studied free-living nematode *Caenorhabditis elegans* and more recently *Ascaris suum,* a non-filarial nematode parasite [17]. While many filarial nematodes are relatively small in size, *Dirofilaria immitis*, the causative agent of canine heartworm disease, is much larger in both length (20–30 cm) and diameter, enabling more accurate dissection of tissues. Here, we describe the first tissue specific transcriptomic profiling of a filarial nematode (*D. immitis)* and its *Wolbachia* endosymbiont. Although RNA levels may imply potential translated protein levels, transcriptomics alone cannot provide any direct information about protein abundance in a tissue. Therefore, we concurrently performed mass spectrometry-based proteome analysis on a number of *D. immitis* tissues in order to corroborate transcriptomic data, expand our understanding of filarial nematode biology and identify tissue specific proteins that may be of interest in filarial nematode drug, diagnostic and vaccine development.

## Methods

### *D. immitis* tissue preparation

Adult *D. immitis* were obtained from the NIH/NIAID Filariasis Research Reagent Resource (FR3) Center for mass spectrometry analysis or purchased from TRS Labs for transcriptomic analysis. All animal procedures were conducted in accordance with IACUC guidelines. Biological replicates for transcriptomic analysis were from two untreated laboratory derived *D. immitis*-infected dogs. Worm dissections were performed using a dissecting microscope on either live or frozen adult *D. immitis* by mounting them on a wax pad and making a long head-to-tail incision using iridectomy scissors. The identity of each tissue piece removed from the worms was confirmed microscopically before tissues were rinsed three times in PBS prior to freezing at −80 °C. Worms were dissected into the following tissues or regions of the body: female body wall (FBW), female uterus (FU), female intestine (FI), female head (FH), male body wall (MBW), male testis (MT), male intestine (MI), male head (MH) and male spicule (MS). The *D. immitis* uterus tissue consists of the entire female reproductive tract, including the intrauterine contents (oocytes, developing embryos and immature microfilariae) and the male testis tissue comprised the entire male reproductive tract including sperm. Likewise, both the *D. immitis* male and female intestine preparations contain the entire nematode digestive tract posterior to the pharynx. The worm head was isolated by making a cut approximately 1 mm posterior to the nerve ring as visualized via stereomicroscope. The female and male head tissues consisted of the entire head including cuticle, body wall, mouth, amphidial neurons, nerve ring with some peripheral nerves as well as the pharynx. Male spicule samples represent the male tale severed from the body approximately 1 mm anterior of the spicule shafts. All *D. immitis* tissues were fast frozen in 1.5 mL graduated round bottom tubes (Eppendorf) and stored at −80 °C prior to RNA or protein isolation.

### Total RNA isolation and library preparation

Tissue samples were homogenized with ceramic beads in CK14 tubes using a Minilys homogenizer (Precellys) and total RNA was extracted by organic extraction using Trizol (Ambion). Samples were treated with DNase I (Ambion) at 37 °C for 30 min before further Trizol extraction and final purification. The RNA integrity, purity and concentration of all samples were assessed on an RNA nano chip using a Bioanalyzer 2100 (Agilent Technologies). In order to capture any *Wolbachia* transcripts, total RNA was used to prepare all RNA-Seq libraries. Samples were prepared for sequencing using the NEBNext® mRNA Library Prep Master Mix Set for Illumina® (New England Biolabs), according to the instructions. Library quality was assessed using a DNA high sensitivity chip on a Bioanalyzer 2100.

### Transcriptome sequencing and bioinformatic analysis

Transcriptomic libraries were sequenced using an Illumina GAIIx sequencer. Data were collected for 50 bp single-end reads. All data was analyzed using a local instance of Galaxy [18–20]. Sequence reads from each tissue sample were first assessed for quality using FastQC (v 1.0.0) [21] and further analyzed using the Tuxedo protocol [22]. RNA-Seq reads from each sample were aligned to the *D. immitis* genome (version 2.2) [14] using TopHat (v. 1.4.1) [23]. Default parameters were used except for the maximum number of alignments to be allowed was set to 40. Reads aligned using TopHat were assembled into transcripts using Cufflinks (v. 1.3.0). Default parameters were used. Cufflinks assemblies from all samples were merged using Cuffmerge (v.1.0.0) and used for differential expression testing using Cuffdiff (v.1.3.0), with the false discovery rate (FDR) set to 0.01.

Similarly, the RNA-Seq reads from each sample were also mapped to the *Wolbachia* from *D. immitis* (*w*Di) genome (version 2.2) [14] using Bowtie [24]. Reads aligned using Bowtie were assembled into transcripts using Cufflinks, then merged with Cuffmerge. Differential expression profiles were determined using Cuffdiff (v. 2.1.1). Default parameters for Cuffdiff were used except the minimum alignment count was set to 2 and FDR set to 0.01.

Hierarchical clustering analysis was performed using Cluster 3.0 [25]. Mapped reads from biological replicates (BAM output files from either TopHat or Bowtie) were first merged then assembled into transcripts using Cufflinks. Normalized FPKM (Fragments Per Kilobase of transcript per Million mapped reads) values were hierarchically clustered using Pearson’s uncentered correlation coefficient with a centroid linkage. Clustered data were depicted graphically (heatmap and dendrogram) using JavaTreeView [25]. Significantly enriched GO terms were identified using the web based Gene Ontology Enrichment Analysis Software Toolkit [26] with the FDR set to 0.1.

### Protein isolation and ms-based proteomic analysis

*D. immitis* tissue samples were lysed in 1 % SDS by sonication (3 times for 30 s) followed by incubation at 65 °C for 30 min with gentle rocking. Samples (100 μL cell lysate) were then prepared for 2D-LC-MS/MS based proteome analysis using the filter aided sample preparation (FASP) kits (Expedion) with modifications to the protocol for simplification (for a complete protocol see [27]). Samples were digested on-filter with 10 μg of Trypsin-ultra Mass Spectrometry Grade (New England Biolabs) overnight at 37 °C. Digested samples were eluted into a clean collection tube via three steps of centrifugation at 14,000 × g for 15 min as follows: Step 1: 80 μL 50 mM ammonium bicarbonate solution; Step 2: 50 μL 0.5 M sodium chloride solution (provided with the FASP kit); Step 3: 170 μL H_2_O with 0.1 % formic acid. The final solution (~300 μL) was split into 2 aliquots per sample and frozen at −20 °C until analysis by 2D-LC-MS/MS. The eluted solution was ready to load onto a 2D-nano LC column without further purification.

The resultant complex peptide mixtures from the individual samples were loaded onto a biphasic C18-SCX (reverse phase-strong cation exchange) (Phenomonex) self-packed nano pre-column (3 cm C18, 3 cm SCX, 150 μm ID) that serves as the first dimension of the 2D-LC system to capture peptides and wash away salts. Once loaded, the column was moved in-line with a U3000 HPLC (Dionex, subsidiary of Thermo Fisher Scientific) split to obtain ~300 nL/min flow rate over the nano-analytical columns. The pre-column was washed with 100 % aqueous solvent followed by an organic solvent gradient (70 % acetonitrile, 0.1 % formic acid) to remove salts and move the peptides to the SCX phase. The pre-column was then attached to a 15 cm × 100 μm C18 front resolving column with an integrated nanospray tip (New Objective Picofrit) packed with Phenomenex Aqua C18. The resolving column was housed in a nanospray source (Proxeon, Thermo Fisher Scientific) attached to a QExactive mass spectrometer (Thermo Fisher Scientific). An automated 22 h two-dimensional LC-MS/MS run was programmed into Xcalibur (Thermo Fisher Scientific) and each sample was analyzed with a separation scheme consisting of eleven salt pulses followed by 2 h C18 separation (for more details on liquid chromatography method see [27]). During each analysis and all sample runs, the QExactive settings were as follows: the normalized collision energy for HCD was 28 eV, a full scan resolution of 70,000 K from 400–1600 m/z, a HCD MS/MS resolution of 17,500 with an isolation width of 3 m/z, and the dynamic exclusion was set at 15 s. Peptides were not excluded based on charge state and 1 microscan for both full and MS/MS scans were acquired. All MS and MS/MS data were acquired in profile mode.

### Proteome informatics

All resultant MS/MS spectra from individual 24 h runs were searched with the Proteome Discoverer V. 1.4 (Thermo Fisher Scientific) and filtered via reverse database searching with maximum false positive rate of 0.5. The Proteome Discoverer settings were as follows: HCD MS/MS, included a fixed modification for carboxyamidomethylated cysteines, a variable modification for urea carbamylation of arginine and lysine residues, fully tryptic peptides only, up to 4 missed cleavages, a precursor mass tolerance of 10 ppm and a fragment mass tolerance of 0.6 Da. Only proteins identified with two fully tryptic peptides from a 22 h run were considered for further analysis. Tandem MS/MS spectra were searched against a combined protein database of *D. immitis* (12,893 entries) and *w*Di (871 entries).

As with the RNA-seq data, hierarchical clustering analysis was performed on the resultant filtered proteome datasets using Cluster 3.0 [25]. Peptide spectral count values were hierarchically clustered using Pearson’s uncentered correlation coefficient with a centroid linkage. Clustered data were depicted graphically (heatmap and dendrogram) using JavaTreeView [25]. Significantly enriched GO terms were identified using the web based Gene Ontology Enrichment Analysis Software Toolkit [26] with the FDR set to 0.1.

## Results and discussion

### *D. immitis* tissue transcriptome overview

Over 30 million single-end 50 bp reads were generated using total RNA from various *D. immitis* tissues, ~84 % of which mapped with high quality to the *D. immitis* reference genome (Table 1). The remaining ~16 % of reads are likely composed of *Wolbachia* transcripts and contaminants (dog and human). Of the mappable reads, approximately 87 % were uniquely mapped to the *D. immitis* genome, while the remaining ~12.3 % of reads (including ribosomal RNA) mapped to multiple locations within the genome. Importantly, *D. immitis* gene model coverage and FPKM (Fragments Per Kilobase of transcript per Million mapped reads) distribution did not vary significantly among the different tissues (Additional file 1: Figure S1A). Close correlation was observed between biological replicates from the *D. immitis* tissues, except the uterus (Additional file 1: Figure S1B-F). Interestingly, the uterus displayed the highest transcriptional variability between biological replicates (R^2^ = 0.73), possibly due to the different infection ages of the worms in each sample (one sample was 1 year at harvest, the other sample was 4 years old at harvest).

**Table 1:** Overview of *D. immitis* tissues analyzed in current study

Sample	Total reads (millions)	High quality million mapped reads	High quality million mapped reads (combined)	Average coverage (FPKM)/transcript	% of all predicted *D. immitis* transcripts detected	Number of *D. immitis* proteins detected		
FBW	1.8	1.5	2.6	37.5	55	1800		2408
						1912		
	1.3	1.1						
						1324		
FU	1.9	1.6	3.8	66.8	74	3341		
	2.5	2.2						
FH	1.5	1.3	2.6	25.4	56	1100		
	1.5	1.3						
FI	1.8	1.5	3.3	38.2	60	202		
	2.1	1.8						
MBW	7.4	5.1	8.5	70.1	64	-		
	5.1	3.4						
MI	7.6	4.5	4.5	58.7	69	-		
MT	7.4	4.7	4.7	61.8	76	-		
MS	-	-	-	-	-	35		
MH	-	-	-	-	-	1183		

*D. immitis* tissues (*FBW* female body wall, *FU* female uterus, *FH* female head, *FI*female intestine, *MBW* male body wall, *MI* male intestine, *MT* male testis, *MS* male spicule, *MH* male head) analyzed by RNA-seq and/or MS-based proteomics. Values from each biological replicate (tissue samples from different worms) analyzed by each method are presented. Technical replicates were pooled prior to further analysis. Total number of unique proteins identified are presented for each tissue (bracketed for FBW biological replicates)

Reads from the *D. immitis* tissues were also mapped to the *Wolbachia* endosymbiont (*w*Di) genome (Table 2). As anticipated, significantly fewer reads mapped to *w*Di genes (Table 2) than to *D. immitis* genes (Table 1) in each tissue: on average ~9 % of sequenced reads (ranging from 0.004 to 25 % depending on the tissue) mapped to *Wolbachia*. A relatively high number of *w*Di transcripts (~90 %) were detected by sequencing total RNA from *D. immitis* hypodermal tissues (female head, female body wall and male body wall), fewer transcripts were detected in the *D. immitis* uterus (~70 % of transcripts) and as expected very few *Wolbachia* transcripts were detected in the intestine and testis samples (≤10 %) (Table 2).

**Table 2:** *Wolbachia* Transcriptome and Proteome Coverage in *D. immitis* Tissues

Sample	*w*Di mapped reads	*w*Di mapped bases (millions)	Average coverage (FPKM)/transcript	% of all predicted *w*Di transcripts detected^a^	Number of *w*Di proteins detected		
FBW	304,004	21.9	229.5	90	134		161
					92		
	443,364	31.9					
					59		
FU	15,109	1.1	922.0	69	33		
	82,897	6.0					
FH	308,412	22.2	216.3	86	34		
	202,058	14.5					
FI	1152	0.08	1627.3	7.1	0		
MBW	440,851	22.0	621.5	97	-		
	337,307	16.9					
MI	1111	0.05	1536.2	10	-		
MT	264	0.01	1367.9	8.3	-		
MS	-	-	-	-	0		
MH	-	-	-	-	62		

^a^ Based on number of transcripts expressed (FPKM >0) per life cycle stage. Values from each biological replicate (tissue samples from different worms) analyzed by each method are presented. Values from each biological replicate (tissue samples from different worms) analyzed by each method are presented. Technical replicates were pooled prior to further analysis. Total number of unique proteins identified are presented for each tissue (bracketed for FBW biological replicates)

**Fig. 1:**
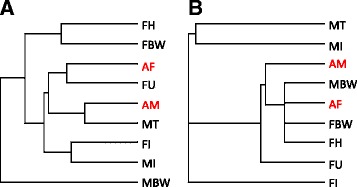
Global comparison of *D.immitis* tissue transcriptomes. Hierarchical clustering reveals similarities between the transcriptional profiles of *D. immitis* (**a**) and *w*Di (**b**) from different tissues (*FH* female head, *FBW* female body wall, *FU* female uterus, *MT* male testis, *FI* female intestine, *MI* male intestine, *MBW* male body wall) and whole adult worms (*AF* adult female, *AM* adult male, from [16], shown in *red*)

Inclusion of recently published whole-worm adult male (AM) and adult female (AF) transcriptomes [16] in the cluster analysis of the *D. immitis* tissue transcriptomes revealed the adult worm transcriptional profiles were most similar to their respective sex organs (testis or uterus) (Fig. 1a). Unlike the *D. immitis* AM and AF transcriptomes, which are dominated by the testis or uterus gene expression profiles, the AM and AF *Wolbachia* transcriptomes were most similar to the body wall and head tissue (which includes body wall) transcriptomes (Fig. 1b).

### Tissue-associated expression

**Fig. 2:**
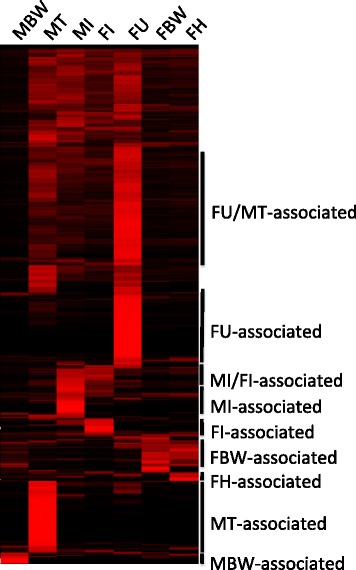
Comparison of *D. immitis* gene expression profiles between *D. immitis* tissues. Data from biological replicates were combined prior to clustering. The color scale ranges from *black* (no expression) to *red* (very high expression). Since Additional file 2: Dataset S1 lists all currently annotated *D. immitis* protein coding genes, only genes expressed in at least one tissue are shown (*n* = 11,640). Each gene is represented by a single row of *boxes*. Tissue-specific clusters used for tissue-specific functional analysis are indicated (*black bars*)

**Fig. 3:**
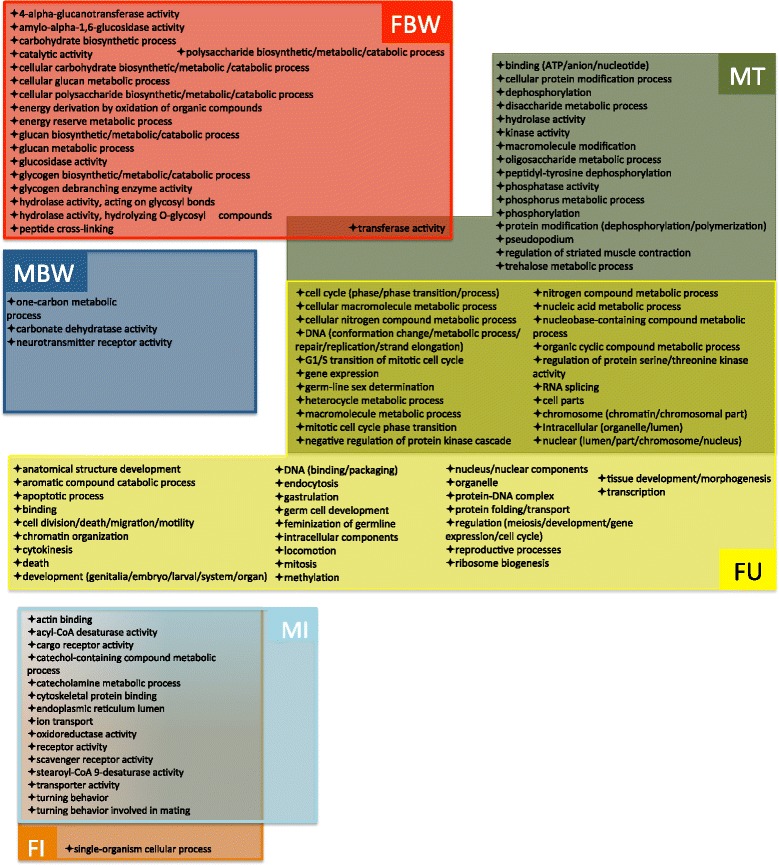
GO terms significantly enriched in *D. immitis* tissues. Overlapping *colored boxes* represent GO terms enriched in both tissues. Due to the large number of GO terms enriched within the uterus sample, many GO terms were grouped together under general terms. The complete list of tissue-enriched GO terms is available in Additional file 3: Table S1

To identify groups of genes preferentially transcribed in specific tissues, hierarchical clustering was performed using the FPKM values of all *D. immitis* protein-coding genes expressed (Additional file 2: Dataset S1) in the seven tissues examined by RNA-seq. The resulting heatmap indicates sets of genes expressed predominantly within a tissue or groups of tissues (MI/FI, FU/MT) (Fig. 2). These tissue-associated gene sets were then classified using GO terms which relate genes to biological function. GO enrichment analysis revealed that transcription of genes associated with carbohydrate (glycogen) processes and collagen formation (peptide-crosslinking) was functionally enriched in the FBW-associated gene set (Additional file 3: Table S1, Fig. 3). Although the FH and FBW samples display similar transcriptional patterns (Fig. 2), no functional enrichment was observed for the small subset of uniquely FH-associated transcripts. Interestingly, neurotransmitter activity and carbon utilization (one-carbon metabolic processes and carbonate dehydratase activity) were enriched among transcripts associated with the MBW (Additional file 3: Table S1, Fig. 3). Perhaps unsurprisingly, functions such as transport and receptor activity were enriched among transcripts associated with the nematode intestine (MI/FI-associated), as were a number of functions seemingly unrelated to common intestinal functions (catecholamine metabolic processes, cytoskeletal/actin binding and turning behavior involved in mating) (Additional file 3: Table S1, Fig. 3). The largest gene cluster was expressed predominantly in the sex organs of both males and females (FU/MT-associated). Within this cluster, GO analysis revealed enrichment of genes involved in the cell cycle/mitosis, DNA repair/replication, RNA processing, gene expression and germ-line sex-determination (Additional file 3: Table S1, Fig. 3). Additionally, an unshared cluster of genes was also specifically associated with each sex organ (MT or FU). GO enrichment of these gene sets uncovered additional functions uniquely associated with the uterus (regulation of metabolic processes, DNA binding and protein methyltransferase activity) and testis (phosphorylation/dephosphorylation, trehalose metabolic processes, striated muscle contraction and pseudopodium) (Additional file 3: Table S1, Fig. 3). Many of the GO terms enriched within the *D. immitis* uterus and testis were previously identified as functions enriched in the ovary and testis of *A. suum* [17].

Many of the previously predicted enriched functions within adult worm transcriptomes [16] appear to arise from enriched functions in specific tissues, especially the testis. Indeed, 35 out of 74 GO terms previously identified as enriched within either *D. immitis* adult males or females were enriched within a specific tissue (Additional file 4: Table S2). Thirty GO terms enriched among male testis transcripts were also significantly enriched in the whole-worm AM transcripts [16], further suggesting the AM transcriptome is strongly influenced by the testis transcripts (Additional file 4: Table S2). Furthermore, the enrichment was typically more significant in the testis tissue than whole-worm adult male transcriptome (Additional file 5: Figure S2). Many of these GO terms (MT and AM-associated) were also enriched within the female uterus sample (Additional file 4: Table S2), however, out of the 15 GO terms enriched in the *D. immitis* whole-worm adult female transcriptome none were shared with the *D. immitis* uterus transcriptome.

As expected, more *w*Di transcripts were detected in nematode tissues known to contain *Wolbachia* (FBW, FH which includes body wall tissue, FU and MBW, Table 2). Nonetheless, transcripts of *w*Di genes were identified in every tissue transcriptome, including the testis and intestine samples. However, due to the especially low abundance of *w*Di transcripts in the FI samples, mapped reads for the two FI biological replicates were merged prior to transcript assembly (Cufflinks) and differential expression testing (Cuffdiff). Interestingly, much lower *Wolbachia* transcript coverage was observed in the *D. immitis* female uterus than the female body wall (69 vs. 90 %, Table 2) and may be due to lower *Wolbachia* numbers per cell in the uterus tissue, high levels of nematode transcription (associated with rapid cell division) in uterus tissue (Table 1) or *Wolbachia* within the body wall tissue may be more transcriptionally active than *Wolbachia* within the uterus tissue.

Hierarchical clustering of *Wolbachia* transcriptomic data from *D. immitis* tissues also revealed clusters of *Wolbachia* genes preferentially transcribed in certain tissues (described in further detail below) (Additional file 6: Figure S3, Additional file 7: Table S3), however, no significant GO term enrichment was observed within any of these tissue-associated gene clusters.

### Gender differences

#### *D. immitis* body wall

Through its involvement in protein secretion as well as host immune evasion, the body wall and cuticle of a filarial nematode comprise the critical interface of the parasite-host interaction. Only 113 transcripts were differentially expressed between the male and female body wall (Additional file 8: Table S4). Approximately 50 % (*n* = 59) of these genes were upregulated in the MBW and include transcripts for major sperm proteins (potentially caused by contaminating transcripts from ruptured testis during dissection), cuticular collagens, an RNA helicase, a matrix metalloproteinase-like protein, a zinc finger-like protein and *egl-9*, a dioxygenase necessary for normal muscle function in *C. elegans* [28].

Transcripts for a number of proteins involved in muscle function, including myosin, actin binding proteins such as kettin or *ketn-1*, titin family member *ttn-1*, troponin t, ph domain-containing protein 4, lim domain and actin-binding protein 1 and the alpha-actinin homologue *atn-1* were among the remaining 54 genes upregulated in FBW. Additionally, *unc-89* and *unc-22* (also known as twitchin), located in muscle A-bands of striated muscle and required for normal muscle function in *C. elegans* [29, 30], were upregulated in the *D. immitis* FBW. Other transcripts upregulated in the FBW compared to MBW include those for filarial antigen, a NAALADase ii peptidase protein, as well as LDL and HDL binding proteins. Additionally, the *him-4* (high incidence of males) transcript that encodes a hemicentin was upregulated in the *D. immitis* FBW. Hemicentin is an evolutionarily conserved extracellular matrix protein secreted by skeletal muscle and the gonad that is involved in cell adhesion, migration and tissue attachment to the epidermis [31, 32].

#### *Wolbachia* expression differences in the body wall of *D. immitis*

Only 33 *Wolbachia* transcripts were differentially expressed between the female and male body wall samples (Additional file 9: Table S5). However, all of these transcripts were upregulated in the MBW. Among these transcripts were five hypothetical proteins, as well as *w*Di genes involved in heme biosynthesis (*hemB*), purine biosynthesis (*purS*) and the type IV secretion system (T4SS) (*VirB6*). In agreement with the differential gene expression results (Additional file 9: Table S5), hierarchical clustering revealed MBW-associated *w*Di genes were involved in purine, heme and folate metabolism as well as secretion (Sec translocase) (Additional file 6: Figure S3, Additional file 7: Table S3). Although all genes in these biosynthetic and transport pathways were not upregulated in the male body wall, these results may suggest important biological differences in the *Wolbachia* symbiosis in males (which only contain *Wolbachia* in the lateral cords) and females (which contain *Wolbachia* in the ovaries and developing embryos as well as the lateral cords).

Although no *Wolbachia* genes were significantly upregulated in the *D. immitis* female body wall compared to the male body wall, hierarchical clustering of *w*Di expression data revealed four hypothetical proteins, one gene involved in isoprene biosynthesis and a gene involved in gluconeogenesis were present among the 9 FBW-associated *Wolbachia* genes (Additional file 6: Figure S3, Additional file 7: Table S3).

#### *D. immitis* intestine

More gender-associated differences were observed when comparing the *D. immitis* intestinal transcriptomes (*n* = 337) than the body wall transcriptomes (*n* = 113) (Additional file 8: Table S4). Among the FI upregulated transcripts were *eat-18*, *nhr-49* and *nhr-31*. Originally identified as a feeding-defective mutation in *C. elegans* [33], *eat-18* is required for nicotinic excitation of the pharyngeal MC neuron and thus, rapid pharyngeal pumping [34]. Of the two nuclear hormone receptors, *nhr-49* appears to function as a key regulator of nutrient response and fat metabolism by influencing the expression of other metabolism related genes in *C. elegans* [35, 36]. Transcription factor activity of the other nuclear hormone receptor upregulated in FI, *nhr-31*, is required for proper growth, development, and function of the excretory cell in *C. elegans* [37].

Notable among the transcripts upregulated in male intestine (compared to female intestine) were transcripts for a tubby protein homolog and *lam-3*. First identified in mice [38], when mutated, tubby proteins cause delayed onset obesity. Later studies in *C. elegans* confirmed the role of tubby proteins in fat and carbohydrate metabolism [39]. Required for normal tissue development (including pharyngeal development), laminins, including *lam-3* are expressed in the pharynx and hypodermis, and also weakly in the intestine of *C. elegans* [40]. Additionally, a number of potential transcription factors including zinc finger proteins, kruppel like factor, ef hand containing proteins and calmodulin-dependent calcium sensors were upregulated in the male compared to female intestine.

#### *Wolbachia* expression in *D. immitis* intestine

Not surprisingly, very few *Wolbachia* genes were transcribed in the intestine of either *D. immitis* sex (*n* = 62 transcripts in the female intestine, *n* = 91 in the male intestine) (Table 2). Interestingly, however, of those *Wolbachia* transcripts detected in the intestine samples, expression levels (FPKM values) were relatively high compared to the other tissues tested (Additional file 6: Figure S3).

Only 15 *w*Di transcripts were expressed in both intestinal tissues, including four hypothetical proteins. Interestingly, in support of previous *Wolbachia* transcriptomic data from the cattle parasite *Onchocerca ochengi* that suggests *Wolbachia* may provide energy (ATP) to its host in certain tissues [41], transcripts for a number of *w*Di proteins/enzymes involved in energy production (including cytochrome C, ATP synthase β chain and isocitrate dehydrogenase) were detected in both male and female *D. immitis* intestine. Hierarchical clustering of the *Wolbachia* gene expression data from the *D. immitis* tissues (Additional file 6: Figure S3) revealed a number of *w*Di proteases among the 42 genes preferentially transcribed in the FI, as well as a phosphate transporter and a heme exporter (CcmB) (Additional file 7: Table S3). Prominent among the MI-associated *Wolbachia* transcripts were genes involved in secretion (T4SS, Sec and Tat translocase components), as well as genes involved in isoprene, riboflavin and peptidoglycan biosynthesis (Additional file 7: Table S3). Exactly how these transcripts were detected in a tissue shown to be completely devoid of *Wolbachia* [5] remains unclear; however, it is possible these *Wolbachia* transcripts were exported from other nematode tissues. Notably, no *Wolbachia* peptides were detected by mass spectrometry in the *D. immitis* female intestine (see below).

#### *D. immitis* reproductive tissues

The reproductive tissues, i.e.*,* testis and uterus, are a significant source of excretory-secretory products of filarial nematodes [42], known to elicit a significant immune response in filarial nematode infected animals: serum from *Acanthocheilonema viteae*-infected rodents strongly reacts with both male and female reproductive tissues [43]. Previous cluster analysis of four nematode proteomes (*B. malayi*, *C. elegans*, *Trichinella spiralis* and *A. suum*) with the predicted *D. immitis* nuclear proteome (version 1.3) identified 850 conserved proteins uniquely shared by *D. immitis* and *B. malayi*, but lacking in the three other nematode species [14]. Previous BLASTP analysis with the improved version 2.2 *D. immitis* proteome removed redundancies and yielded 834 predicted proteins which may represent common filarial nematode targets shared between *D. immitis* and *B. malayi* [16]. Assessment of tissue-specific transcription of these 834 gene products is listed in Additional file 8: Table S4. Many of these 834 genes (*n* = 523) displayed tissue-associated transcriptional patterns. Of these 523 *D. immitis* genes with tissue-associated transcriptional patterns, over 80 % (*n* = 421) were associated with a reproductive tissue (FU, MT or FU/MT) of *D. immitis.* Over 200 of these transcripts expressed in the FU were confirmed by proteomics (see below). It is clear the reproductive tissues express transcripts and secrete proteins that may serve to target filarial nematode reproduction in a transmission blocking approach to disease control. Perhaps unsurprisingly, the pairwise comparison of the uterus and testis sample produced the largest number (*n* = 2998, Additional file 9: Table S5) of differentially expressed genes compared to any other tissue comparison by gender (FBW v. MBW or FI v. MI).

##### Genes upregulated in the *D. immitis* uterus

Of the nearly 3000 transcripts differentially expressed between the *D. immitis* uterus and testis tissues, 2300 are upregulated in the uterus. A number of notable transcripts were expressed in the uterus tissue, but not expressed at all in the male testis. These include the follicle stimulating hormone receptor, *nhr-23*, *unc-7*, *unc-9*, *unc-62*, *odr-3*, a zona pellucida-like domain containing protein, GABA and dopamine transporters, riboflavin kinase and phosphomevalonate kinase. A member of the nuclear hormone receptor family of transcription factors [44], *nhr-23* (through the regulation of collagen and hedgehog related genes [45]) has been shown to be critical to all four larval molts in *C. elegans* [46] and may therefore represent a transcript that is highly expressed in the intrauterine microfilariae of *D. immitis* in preparation for future molts. The three uncoordinated (unc) proteins expressed in the uterus but not expressed in the testis (*unc-7*, *-9* and *-62*) are all implicated in female reproductive development in *C. elegans*: *unc-7* and *unc-9*, both gap junction forming innexins, are required for normal egg laying [47, 48] and *unc-62* is necessary not only for vulval morphogenesis but also posterior embryonic morphogenesis [49]. Curiously, another gene expressed in the *D. immitis* uterus tissue but not in the testis was *odr-3*, which encodes for a G-protein subunit typically expressed in neurons and required for normal chemotaxis in *C. elegans* [50].

Among transcripts expressed in both the uterus and testis, but upregulated in the female reproductive tract were transcripts for doublesex, *mab-3*-related transcription factor 1, transcription factor bigmax, apoptosis regulatory protein, placental protein 11, DNA damage-binding protein 1, chitin synthase 1 (*chs-1*), cannabinoid receptor 1, *gld-2, nab-1*, *unc-112* related protein and a number of nuclear hormone receptors (*nhr-6, nhr-7a*, *nhr-14*, *nhr-41* and *nhr-91*). Of these nuclear hormone receptors, *nhr-6*, an orthologue of the *Drosophila melanogaster E75* gene which plays a role in molting, was previously shown to be highly expressed in *D. immitis* adult females compared to adult males [51], while *nhr-14* controls gene expression in response to estrogen in *C. elegans* [52]. Other transcripts upregulated in the *D. immitis* uterus have been implicated in germ line (*let-49* [53], *sax-7* [54], *sel-12* [55]), oocyte (*mep-1* [56], *daz-1* [57]) or embryonic (chitin synthase 1 [58], *nrf-6* [59], *die-1* [60], *gei-17* [61]) development in *C. elegans*. Interestingly, two of these genes (chitin synthase I and *nrf-6*) upregulated in the uterus compared to the testis were predicted to be among the top 40 drug targets for the related filarial nematode, *B. malayi* [62].

##### Genes upregulated in the *D. immitis* testis

A total of 698 transcripts were upregulated in the male testis compared to the female uterus, nearly 80 % of which (*n* = 471) displayed no expression in the uterus (Additional file 9: Table S5). Perhaps not surprisingly, the list of genes upregulated in the testis (which contains sperm) compared to the uterus includes a number of major sperm proteins. Also upregulated in the *D. immitis* testis were transcripts for *fog-3*, *gld-1*, *spe-4* and *spe-9*. In *C. elegans*, *fog-3* is most highly expressed in the L3, L4 and early adult stages and is essential for spermatogenesis [63]. Additionally, *gld-2*, along with *gld-3*, activates *gld-1* to regulate entry into meiosis during germ cell development [64]. In the *D. immitis* tissues, *gld-2* was expressed in both testis and uterus, but was upregulated in uterus, whereas *gld-1* was only transcribed in the testis. Interestingly, in the absence of *gld-1*, *gld-2* is still able to promote meiosis [64], suggesting that *gld-2* likely has other unidentified targets (particularly in the female germline where *gld-1* is not transcribed but meiosis is occurring). Both *spe-4* and *spe-9*, which were upregulated in the testis, are critical to spermatogenesis in *C. elegans* [65]. While *spe-4* is involved in proper partitioning of cytoplasmic components during spermatogenesis [66], *spe-9* mediates oocyte fertilization likely through an adhesive function or signaling event [67]. Also prominent among transcripts upregulated in the testis compared to the uterus were msp-domain containing proteins, a designated sperm-specific sodium proton exchanger, phosphatases and kinases, as well as and a number of hypothetical proteins conserved between *D. immitis* and related filarial nematodes *B. malayi* and *L. loa*.

#### *Wolbachia* expression in *D. immitis* uterus and testis

Among 86 *Wolbachia* genes preferentially transcribed in the *D. immitis* uterus (Additional file 7: Table S3) were genes involved in purine and pyrimidine biosynthesis, heme biosynthesis, isoprene biosynthesis, energy production, fatty acid biosynthesis and secretion (components of the Sec translocation system). Since *Wolbachia* are absent in the male germ line of filarial nematodes [5], very few *Wolbachia* transcripts were detected in the testis tissue of *D. immitis* (Table 2). Interestingly however, transcripts suggested to be involved in the symbiosis between filarial nematodes and *Wolbachia* were among the *w*Di MT-associated transcripts including those associated with secretion systems (components of the T4SS, the type 1 secretion system and the Sec translocase), purine and peptidoglycan biosynthesis as well as heme metabolism (Additional file 7: Table S3).

#### *D. immitis* female head tissue expression

The female head tissue included cuticle, body wall, mouth, amphidial neurons, nerve ring with some peripheral nerves as well as the pharyngeal muscle. Although no GO terms were enriched among the FH-associated transcripts (Fig. 2), nearly 300 transcripts were upregulated in the *D. immitis* female head tissue when compared to other female tissues examined (FBW, FI or FU, Additional file 9: Table S5). These head upregulated transcripts include those potentially linked to neuronal activity such as *ptc-3* [68] and *slo-2* [69, 70]. Muscle-associated transcripts for *dim-1*, *unc-87* and *unc-89* (Additional file 9: Table S5) were upregulated in FH compared to FI or FU, but were much higher in the FBW tissue than the head tissue, suggesting these highly expressed FH transcripts likely originate from the body wall muscle and not the pharyngeal muscle.

#### *Wolbachia* expression in *D. immitis* female head

Previous immunofluorescence staining confirmed the absence of *Wolbachia* in nerve ring of adult *B. malayi* [5]. Therefore it is not surprising that the *Wolbachia* transcriptome of the *D. immitis* female head closely resembles the *Wolbachia* transcriptome of the female body wall tissue (Additional file 6: Figure S3), since transcripts detected in the FH likely originate from the hypodermal cords in the cephalic region.

### Tissue comparisons

#### *D. immitis* body wall versus intestine

Vaccination of laboratory animals with intestinal antigens from parasitic nematodes has conferred partial immunity to subsequent parasite infection [71–73], making intestinal antigens attractive vaccine targets. A thorough comparison of the *D. immitis* body wall and intestine transcriptomes may identify potential so-called ‘hidden antigens’ present in the intestine. Interestingly, fewer differences in gene expression were observed when comparing the female body wall and female intestine (387 differentially expressed genes) than the male body wall and male intestine (1110 differentially expressed genes) (Additional file 9: Table S5).

#### Genes Upregulated in the *D. immitis* Female Intestine

Of the genes differentially expressed between the *D. immitis* FBW and FI samples, nearly 70 % (*n* = 264) were upregulated in the intestine and could potentially represent ‘hidden antigens’ (Additional file 9: Table S5). Among these transcripts are *gei-4*, jun transcription factor homolog family member (*jun-1*), *daf-16*, *hlh-30, pha-4* and *unc-83*. Concordant with their expression in the intestine, it was recently discovered in *C. elegans* that transcription factors hlh-30, jun-1 and daf-16 play a role in mediating fasting-induced longevity by inducing transcriptional changes and regulating proteolysis [74, 75]. Another transcription factor expressed in the female intestine but not body wall, pha-4, is required for pharynx development in *C. elegans* [76]. Although interesting, these intestine-associated transcription factors are likely unsuitable vaccine targets since they are probably not present on the surface of the intestine.

Other transcripts upregulated in the female intestine compared to the female body wall include: *fat-3*, *mdt-15*, *cyn-5*, *lap-1* and *mrp-5*. In *C. elegans*, both *fat-3* and *mdt-15* are critical to fatty acid metabolism: *mdt-15* coordinates a number of metabolic factors that regulate the expression of fatty acid desaturase genes, such as *fat-3* [77, 78]. A type B cyclophilin encoded by *cyn-5*, a leucine aminopeptidase encoded by *lap-1* and the multi-drug resistance protein encoded by *mrp-5* have all been shown to be expressed in the intestine of *C. elegans* [79–81]. Interestingly, detailed analysis in *C. elegans* revealed that mrp-5 serves as a critical heme transporter that exports intestinally absorbed heme to other tissues [81].

##### Genes Upregulated in the D. immitis Male Intestine

Of the genes differentially expressed between the *D. immitis* MBW and MI, ~55 % (*n* = 613) were upregulated in the intestine (Additional file 9: Table S5). Notable among these transcripts are *gei-4*, *pha-4*, *fat-3*, *mdt-15* and *mrp-5* (also upregulated in FI compared to FBW). Other transcripts upregulated in the *D. immitis* male intestine compared to the male body wall include *exp-2*, *nas-4* and *itr-1*. The *exp-2* gene is required for normal pharyngeal pumping in *C. elegans* by encoding for a potassium channel that allows the pharynx muscles to repolarize quickly [82]. Expressed in the pharynx and intestine of *C. elegans* [83], nas-4 is a zinc metalloproteinase that likely plays a role in nematode digestion. The inositol 1,4,5-triphosphate receptor encoded by *itr-1* is highly expressed in both the pharynx and intestine of *C. elegans* [84] and appears to play a role in pharyngeal and intestinal pumping [85].

#### *D. immitis* body wall versus reproductive organs

A similar number of differences in gene expression are observed when comparing the *D. immitis* female body wall to uterus tissue (1555 differentially expressed genes) and the *D. immitis* male body wall to testis tissue (1642 differentially expressed genes).

Of the differentially expressed genes between the FBW and FU, only 17 % (*n* = 268) are upregulated in the body wall (Additional file 9: Table S5). These transcripts include a number of muscle proteins (actin/myosin), collagens, filarial antigen, galectins and lectin binding proteins as well as transcripts for *him-4*, *alp-1*, *unc-22*, *unc-87*, *unc-89*, *deb-1*, *ketn-1* and *ttn-1*. Importantly, *alp-1, unc-87*, *deb-1, ketn-1* and *ttn-1* have all been associated with the body wall muscle structure and/or function in *C. elegans* [86–92].

The remaining 83 % (*n* = 1287) of transcripts differentially expressed between the *D. immitis* FBW and FU are upregulated in the uterus tissue (Additional file 9: Table S5). Not surprisingly, many of these transcripts (*die-1*, *gei-17*, *let-49*, zona pellucida-like protein, *gld-2*, *sax-7*, *nhr-7a*, *nhr-23*, *unc-7*, *unc-9* and *unc-62*) were also upregulated in the female uterus compared to the male testis. Interestingly, *gei-4* and the transcription factor *jun-1*, which were both upregulated in the female intestine when compared to FBW (see above), are upregulated in the female uterus as well (when compared to the female body wall). In addition to its role in mediating fasting-induced transcriptional changes in the intestine, jun-1 also regulates phospholipase C expression, thereby affecting inositol triphosphate levels which in turn affect ovulation [93]. Other noteworthy transcripts upregulated in the uterus compared to the female body wall were histones, *apr-1*, the lipocalin encoding genes *lpr-4*, *lpr-5* and *lpr-6*, a wnt family protein, the nuclear transport factor *ntf-2* and the mucin-like protein encoded by *let-653*.

Nearly 40 % (*n* = 656) of the genes differentially expressed between the *D. immitis* MBW and MT were upregulated in the body wall (Additional file 9: Table S5). As with the female body wall, these transcripts include muscle proteins such as actin, myosin, collagens as well as filarial antigen and galectins. Additionally, transcripts for *unc-87*, *unc-95*, *mpz-1* and *ttr-5* are upregulated in the male body wall compared to the testis. As mentioned above, *unc-87* is critical for muscle development [87, 88]. The muscle LIM domain containing protein encoded by *unc-95* is required for locomotion and organization of thick and thin filaments in body wall muscle [94]. Interestingly, the *ttr-5* gene encodes a transthyretin-like protein, which was among the most abundant proteins detected in a recent analysis of the excretory-secretory products of another filarial nematode, *Litomosoides sigmodontis* [42].

Many of the transcripts upregulated in the *D. immitis* MT compared to the MBW, including *gei-4*, *pha-4*, *gld-1, unc-7* and *unc-9* have been described in detail above. Additionally, transcripts for a posterior sex combs protein, trehalase, msp domain containing proteins, a dad (defender against death) family protein and *ssp-35* (sperm specific family protein, class P) were upregulated in the testis compared to the male body wall.

#### Comparison of *Wolbachia* expression in *D. immitis* female body wall and uterus

A thorough comparison of *Wolbachia* expression profiles within the germ line and the somatic cells (hypodermis of the body wall) may provide clues as to the varying role of *Wolbachia* within the different nematode tissues inhabited by the endosymbiont. A total of 121 *Wolbachia* transcripts were differentially expressed between the FBW and FU tissues, a large proportion of which (~91 %) were upregulated in the uterus (Additional file 10: Table S6) despite the fact that lower *Wolbachia* transcript coverage was observed in the *D. immitis* female uterus (69 %) than the female body wall sample (90 %) (Table 2). Only 11 *w*Di transcripts were upregulated in the female body wall. These include transcripts for a component of the Sec translocase, components of the T4SS as well as the heme (*hemC*) and pyrimidine biosynthesis pathways (Additional file 10: Table S6), all of which have been previously implicated in the symbiotic relationship between filarial nematodes and *Wolbachia*. Notable among the *Wolbachia* transcripts upregulated in the *D. immitis* uterus include transcripts for a number of other steps in the heme biosynthetic pathway (*hemB*, *hemD*, *hemE*), the T4SS (*VirB9* and *VirB11*) and the Sec translocase (*SecB* and *ffh*) (Additional file 10: Table S6). Additionally, *RibA,* the first gene in the pathway leading to riboflavin synthesis, a putative *Wolbachia*-nematode shared metabolite, was another significantly upregulated *Wolbachia* transcript in the *D. immitis* uterus compared to the female body wall.

Our comparison of the *w*Di transcriptomes from the *D. immitis* uterus and female body wall strongly echoes previous findings that found transcripts involved in translation and DNA replication were upregulated in the germ line *Wolbachia* transcriptome from *O. ochengi* (*w*Oo) in comparison to that of the somatic transcriptome [41]. Notably in terms of translation, elongation factor Tu, 3 ribosomal proteins and polypeptide deformylase were all upregulated in *w*Oo germ line transcriptome compared to the somatic transcriptome. Likewise, we find *w*Di transcripts for elongation factor Ts, 7 ribosomal proteins, a peptide deformylase and a number of tRNA synthetases are all upregulated in *D. immitis* uterus tissue compared to the female body wall tissue (Additional file 10: Table S6). Additionally, *Wolbachia* transcripts related to DNA replication and repair including a uracil DNA glycosylase and DNA recombination proteins RmuC and MutL were upregulated in the *D. immitis* germ line tissue (uterus) compared to the somatic tissue (female body wall). As previously suggested [41], these *Wolbachia* transcripts associated with transcription and DNA replication that are upregulated in the germ line tissue may be related to the fact that prior to fertilization, *Wolbachia* appears to divide rapidly in the germ line of female filarial nematodes [5]. Interestingly, two *Wolbachia* endonuclease transcripts, endonuclease III and Nuc endonuclease, are also upregulated in the *D. immitis* uterus tissue. Since most filarial nematodes are unable to synthesize purines and pyrimidines *de novo*, it is possible that *Wolbachia* endonucleases are recycling nucleotides in order to supplement the host nucleotide pool within the rapidly dividing nematode germ line. Alternatively, *Wolbachia* may be utilizing the nucleotides for their own propagation within the germ line.

### *D. immitis* tissues proteome

#### Overview

**Fig. 4:**
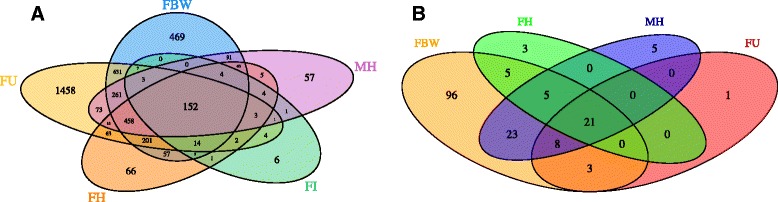
Venn diagrams illustrating *D. immitis* (**a**) and *w*Di (**b**) proteins detected in *D. immitis* tissues. **a** Since only 35 *D. immitis* proteins were identified in the MS tissue sample, the spicule sample was excluded from this figure. However, those 35 proteins were also identified in other tissues and are represented accordingly. **b** Only FBW, FH, MH and FU samples are shown since no *w*Di proteins were detected in the FI or MS sample

Overall, 4162 of the 12,857 annotated proteins or ~32 % of the predicted *D. immitis* proteome were verified by at least two unique peptides from the analysis of six *D. immitis* tissues (FBW: 2408; FU: 3341; FH: 1100; FI: 202; MH: 1183 and MS: 35 proteins) (Table 1, Additional file 11: Dataset S2). Higher protein coverage was obtained with *B. malayi* [95], however that level of coverage was attained using five life cycle stages whereas our data only covers the proteome of adult *D. immitis* tissues. A complete list of the peptide spectrum matching counts for every *D. immitis* protein detected, along with their corresponding gene annotation is provided in Additional file 12: Table S7. Likely due to incomplete lysis of the spicule due to its inherent structural stability, only 35 proteins were detected in this tissue (Additional file 12: Table S7). Because these proteins were also detected in another *D. immitis* tissue, the spicule sample was omitted from further analysis. Only 3.7 % of identified proteins (*n* = 152) were common among the remaining five tissue samples (FBW, FU, FH, FI and MH) (Fig. 4a). Not surprisingly, these shared proteins include common structural and motor proteins (actin/collagens/kinesin), galectins, annexins, redox proteins (thioredoxin peroxidases), heat shock proteins, enzymes (enolase, ATP synthase components, lactate dehydrogenase, pyruvate kinase, protein disulfide isomerase, protein phosphatase) and clathrin, among others (Additional file 12: Table S7). Among the 4162 proteins identified, ~49 % (*n* = 2056) were tissue specific (identified only in one tissue) (Fig. 4a, Additional file 12: Table S7). Perhaps unsurprisingly, the highest number of tissue specific proteins (1458) were identified in the uterus sample (Fig. 4a). Out of the 202 proteins identified in the *D. immitis* female intestine sample, only 6 proteins were intestine-specific. These potential hidden antigens include: aspartic protease sp-1, transmembrane protein 132a, guanine nucleotide-binding protein α-7 subunit gpa-7, cell division protein kinase 5, α-ulin (catenin vinculin related) family member ctn-1 and a hypothetical protein. Interestingly, both gpa-7 and ctn-1 are expressed in the pharyngeal muscle and intestinal muscle of *C. elegans* [96, 97].

Of the predicted 834 filarial-specific proteins (those uniquely shared by *D. immitis* and *B. malayi,* but lacking in *C. elegans*, *T. spiralis* and *A. suum*, Additional file 8: Table S4) [14], 285 were confirmed by proteomics of *D. immitis* tissues. The vast majority of these proteins were detected in the FU sample (*n* = 215), while 136, 67, 66, 11 and 3 of these proteins were detected in the FBW, FH, MH, FI and MS, respectively.

Filarial nematode genomes and corresponding predicted proteomes are riddled with hypothetical and unannotated proteins. Previous proteomic analysis of *B. malayi* life cycle stages was able to confirm the existence of ~2300 hypothetical proteins [95]. Out of the ~5000 hypothetical and unannotated proteins within the *D. immitis* genome, our tissue-specific proteome has validated 744 ‘hypothetical’ proteins (52 annotated as hypothetical proteins and 692 unannotated proteins) (Additional file 12: Table S7). The majority of these hypothetical proteins were detected in the FBW and FU tissues. Intriguingly, 128 of the 744 hypothetical proteins identified herein are putatively filarial-specific antigens (conserved proteins shared by *D. immitis* and *B. malayi*, but lacking in *C. elegans*, *T. spiralis* and *A. suum*, Additional file 8: Table S4) [14].

Hierarchical clustering of *D. immitis* proteins detected in the 6 *D. immitis* tissue samples revealed groups of proteins associated with specific tissues (Additional file 13: Figure S4). GO enrichment analysis of these clusters revealed significantly enriched functions within the FBW, FU, FI and MH-associated protein clusters (Additional file 3: Table S1). Out of the 78 GO terms identified as enriched within the uterus sample by RNA-seq, 48 of those were also identified as enriched by GO analysis of the FU-associated protein cluster, including those involved in cell cycle/mitosis/DNA replication, transcription, metabolic processes, cell parts (chromosome/organelle). In total, over 450 additional uterus-associated GO terms were identified by proteomic analysis. Notable among uterus-associated proteomic GO terms were those involved in anatomical structure development, apoptosis, reproductive processes, chromosome/chromatin organization, cytokinesis, embryo development, protein localization/targeting, gamete generation, gene silencing, mRNA processing and helicase activity.

#### *Wolbachia* proteome in *D. immitis* tissues

Proteomic analysis of the *D. immitis* tissues identified 170 *w*Di proteins (~20 % of the 871 predicted protein encoding genes) based on at least two unique peptides matching a *Wolbachia* protein (Additional file 14: Table S8). A complete list of the peptide spectrum matching counts for every *Wolbachia* protein detected is provided in Additional file 14: Table S8. Likely due to the presence of biological replicates, the vast majority of *Wolbachia* proteins were detected in the FBW sample (Fig. 4b), while surprisingly very few were detected in the uterus tissue. Likely due to the absence of *Wolbachia* in these tissues, no *w*Di proteins were detected in the FI and MS samples (Table 2). Similar to previous proteomics studies on *w*Bm [95] and *w*Oo [41], the most abundant *Wolbachia* proteins detected were chaperonins and heat shock proteins such as GroEL, DnaK, ClpB protein, GroES and surface antigen Wsp (Additional file 14: Table S8).

A number of biosynthetic pathways have been implicated in potential metabolite provisioning between *Wolbachia* and filarial nematodes [11] and may serve as possible *Wolbachia*-specific targetable pathways [16]. Components of many of these pathways, including the heme biosynthesis pathway (3 of 8 enzymes), the purine biosynthesis pathway (6 of 10 enzymes), and the isoprene biosynthesis pathway (1 of 5 enzymes) were detected, mainly in the *D. immitis* female body wall and head tissues (Additional file 14: Table S8). Likely due to the low coverage of the *w*Di proteome, no peptides matching enzymes from *Wolbachia* pathways for the synthesis of pyrimidines (6 enzymes), lipid II/peptidoglycan (9 enzymes), riboflavin (5 enzymes) or folate (5 enzymes) were detected in any *D. immitis* tissue.

Annotation of the *w*Di genome describes 277 of the 871 predicted proteins as hypothetical proteins. Based on our transcriptomic data, 264 of these hypothetical proteins were transcribed (FPKM values >0) in some *D. immitis* tissue (Additional file 10: Table S6). In total, 9 of the 264 transcribed hypothetical proteins were validated as genuine *w*Di proteins by proteomic analysis (Additional file 12: Table S7). Interestingly, some of these ‘hypothetical’ proteins (namely wDi:fig_82301.12.peg.651, wDi:fig_82301.12.peg.439 and wDi:fig_82301.12.peg.677) were also among the 10 most abundant *Wolbachia* proteins detected (Additional file 14: Table S8).

### Comparison of transcriptomics and proteomics

**Fig. 5:**
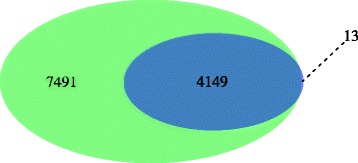
Euler diagram illustrating *D. immitis* genes detected by transcriptomic (*green*) and proteomic (*blue*) methods in *D. immitis* tissues

Often very little correlation exists between transcript and protein abundance [41], because RNA levels may not accurately reflect protein levels since protein levels in vivo are not only dictated by the rate of transcription/translation, but also by the rate of protein degradation. In the 4 *D. immitis* tissues examined by both methods, 11,640 *D. immitis* transcripts and 4162 *D. immitis* proteins were detected. Overall, proteomic analysis of these adult *D. immitis* tissues revealed peptide evidence for nearly 36 % (4149) of transcripts identified in tissues examined by both methods (Fig. 5). Based on individual tissue transcriptomics/proteomics, the female uterus sample had the highest percentage of *D. immitis* transcripts verified within that tissue by mass spectrometry (35 %), followed by the FBW, FH and FI (32, 14 and 3 %, respectively) (Additional file 15: Figure S5A-D). Conversely, only 0.3 % (*n* = 13) of proteins identified lacked any transcriptional evidence in any *D. immitis* tissue examined (Fig. 5), the majority (*n* = 10) of which were detected in the female uterus. Only ~2–7 % of *D. immitis* proteins identified in a tissue lacked transcripts within that same tissue (Additional file 15: Figure S5A-D). These proteins may be transcribed in another tissue then trafficked to the tissues where they were detected by proteomic analysis. Interestingly, 9 proteins identified in the FBW sample lacked detectable transcription in any female tissue, but were transcribed in a male tissue(s).

All 170 *Wolbachia* proteins identified by at least 2 unique peptides in proteomic analysis were transcribed in at least one *D. immitis* tissue. The highest level of *wDi* proteomic coverage of the transcriptome was observed in the *D. immitis* FBW (over 20 % coverage), whereas only 6 and 5 % of transcribed genes were verified in the FU and FH tissues, respectively (Additional file 16: Figure S6A-C). A total of 9 *Wolbachia* proteins were identified by proteomic analysis in a specific tissue that was not confirmed by any transcriptomic evidence in that same tissue (Additional file 16: Figure S6A-C). It is possible these proteins were made either in another *D. immitis* tissue containing *Wolbachia* and then transported/secreted, or alternatively these proteins may have been transcribed during an earlier developmental stage of *D. immitis* prior to localization in tissues seemingly devoid of *Wolbachia*. How these proteins came to be in these tissues while their transcriptional levels were undetectable by RNA-seq remains unclear, but suggests *Wolbachia* proteins and their associated functions may extend beyond the tissue-specific localization of *Wolbachia* within the adult nematode.

## Conclusions

More knowledge is gained with every filarial nematode genome sequence that is analyzed. While the advent of RNA-seq and more sensitive proteomic methods has also been of great value, these methods provide the most significant level of detail when used in concert with one another. Understanding the biology of filarial nematodes and the development of novel therapeutic strategies against them requires a greater spatial understanding of both nematode and *Wolbachia* gene expression and protein abundance. We therefore implemented parallel transcriptomic and proteomic analysis to study filarial nematode tissue-specific transcriptional patterns in order to gain functional information. Such spatial resolution of gene expression can provide greater understanding not only on how that specific tissue may interact with the vertebrate host (or evade the host immune system), but also how *Wolbachia* may differentially contribute to various nematode tissues. Notably, our analysis of *D. immitis* tissues revealed that the reproductive tissue (uterus and testis) transcriptomes most closely correlate with recently published whole-worm transcriptomes, while the *Wolbachia* transcriptome of the body wall tissue most closely correlated with the whole-worm *Wolbachia* transcriptomes. Together, transcriptomics and proteomics of *D. immitis* intestinal tissues highlighted a number of potential hidden antigens. Interestingly, some *Wolbachia* genes upregulated in nematode tissues include transport systems and genes predicted to be involved in the symbiotic relationship with *D. immitis*. Furthermore, the presence of *Wolbachia* transcripts and proteins in *D. immitis* tissues lacking *Wolbachia* suggests that the symbiont transcripts and proteins may traffic to other nematode tissues.

Not only has this study provided a new level of detail to our knowledge of filarial tissue transcription/translation, but also furthered our understanding of how *Wolbachia* endosymbionts may interact with filarial nematodes. For the first time in any filarial nematode, this data in conjunction with the recently published life cycle transcriptome [16], serves as a comprehensive atlas of the temporal and spatial representation of expression of both *D. immitis* and *w*Di genes, including those that may be used to develop future therapeutic strategies.

## Availability of supporting data

The mapped reads (TopHat BAM output files) and FPKM values for assembled transcripts (Cufflinks text output files) are available in the NCBI Gene Expression Omnibus (Series GSE67894).

## Additional files

Additional file 1:
**Figure S1.** Gene coverage and comparison of D. immitis biological tissue replicas. (PDF 25 kb) Additional file 2:
**Dataset S1.** Transcript FPKM values for *Dirofilaria immitis* tissue transcriptome. (XLSX 369 kb) Additional file 3:
**Table S1.** List of over-represented GO terms and their associated p-values for tissue-associated *D. immitis* genes detected by either transcriptomics (red) or proteomics (blue). (XLSX 984 kb) Additional file 4:
**Table S2.** List of over-represented GO terms and their associated p-values for tissue-associated *D. immitis* genes that were also over-represented in the transcriptome of either adult male (AM) or adult female (AF) *D. immitis*. GO terms identified by transcriptomic analysis are highlighted in red while GO terms identified by proteomic analysis are in blue. (XLSX 55 kb) Additional file 5:
**Figure S2.** Significance (p-value) of GO terms shared between D. immitis adult male and testestranscriptomes. (PDF 43 kb) Additional file 6:
**Figure S3.** Clustered transcriptomic data of Wolbachia gene expression profiles in D. immitis tissues. (PDF 21 kb) Additional file 7:
**Table S3.** *D. immitis* tissue-associated *Wolbachia* transcript list based on hierarchical clustering. (XLSX 226 kb) Additional file 8:
**Table S4.** Tissue-associated expression and proteomic detection of the 834 *D. immitis* genes uniquely shared with *B. malayi*. (XLSX 170 kb) Additional file 9:
**Table S5.** List of *D. immitis* genes that are significantly (q < 0.01) differentially expressed between various *D. immitis* tissues. (XLSX 998 kb) Additional file 10:
**Table S6.** List of *Wolbachia* genes that are significantly (q < 0.01) differentially expressed between various *D. immitis* tissues. (XLSX 87 kb) Additional file 11:
**Dataset S2.** *Dirofilaria immitis* and *Wolbachia* proteins identified in *D. immitis* tissues. (XLSX 58 kb) Additional file 12:
**Table S7.** Gene IDs, peptide spectral counts and protein descriptions for all 4162 *D. immitis* proteins detected by mass spectrometry in *D. immitis* tissues. (XLSX 851 kb) Additional file 13:
**Figure S4.** Clustered proteomic data (peptide spectral matches) of D. immitis proteins detected inD. immitis tissues. (PDF 50 kb) Additional file 14:
**Table S8.** Gene IDs, peptide spectral counts and protein descriptions for all 170 Wolbachia proteins detected by mass spectrometry in D. immitis tissues. (XLSX 56 kb) Additional file 15:
**Figure S5.** Euler diagrams illustrating D. immitis genes detected by transcriptomic (green) and proteomic (blue) methods in various female D. immitis tissues: (A) body wall, (B) uterus, (C) head, (D) intestine. (PDF 1275 kb) Additional file 16:
**Figure S6.** Euler diagrams illustrating D. immitis genes detected by transcriptomic and proteomicmethods. (PDF 89 kb)

## Abbreviations

AF: Adult female
AM: Adult male
FASP: Filter aided sample preparation
FBW: Female body wall
FDR: False discovery rate
FH: Female head
FI: Female intestine
FPKM: Fragments Per Kilobase of transcript per Million mapped reads
FU: Female uterus
GO: Gene ontology
LC-MS/MS: Liquid chromatography-tandem mass spectrometry
MBW: Male body wall
MH: Male head
MI: Male intestine
MS: Male spicule
MS/MS: Tandem mass spectrometry
msp: Major sperm protein
MT: Male testis
RP: Reverse phase
SCX: Strong cation exchange
T4SS: Type 4 secretion system
*w*Bm: *Wolbachia* from *Brugia malayi*
*w*Di: *Wolbachia* from *Dirofilaria immitis*
*w*Oo: *Wolbachia* from *Onchocerca ochengi*

## References

[1] Taylor MJ, Hoerauf A, Bockarie M (2010). Lymphatic filariasis and onchocerciasis. Lancet.

[2] World Health Organization (2010). GPELF Progress Report 2000–2009 and Strategic Plan 2010–2020. WHO/HTM/NTD/PCT/2010.

[3] Taylor MJ, Awadzi K, Basanez MG, Biritwum N, Boakye D, Boatin B, Bockarie M, Churcher TS, Debrah A, Edwards G (2009). Onchocerciasis control: vision for the future from a Ghanian perspective. Parasit Vectors.

[4] Osei-Atweneboana MY, Awadzi K, Attah SK, Boakye DA, Gyapong JO, Prichard RK (2011). Phenotypic evidence of emerging ivermectin resistance in *Onchocerca volvulus*. PLoS Negl Trop Dis.

[5] Landmann F, Foster JM, Slatko B, Sullivan W (2010). Asymmetric *Wolbachia* segregation during early *Brugia malayi* embryogenesis determines its distribution in adult host tissues. PLoS Negl Trop Dis.

[6] Taylor MJ, Bandi C, Hoerauf A (2005). *Wolbachia* bacterial endosymbionts of filarial nematodes. Adv Parasitol.

[7] Bandi C, Trees AJ, Brattig NW (2001). *Wolbachia* in filarial nematodes: evolutionary aspects and implications for the pathogenesis and treatment of filarial diseases. Vet Parasitol.

[8] Hoerauf A, Mand S, Adjei O, Fleischer B, Buttner DW (2001). Depletion of *Wolbachia* endobacteria in *Onchocerca volvulus* by doxycycline and microfilaridermia after ivermectin treatment. Lancet.

[9] Hoerauf A, Specht S, Buttner M, Pfarr K, Mand S, Fimmers R, Marfo-Debrekyei Y, Konadu P, Debrah AY, Bandi C (2008). *Wolbachia* endobacteria depletion by doxycycline as antifilarial therapy has macrofilaricidal activity in onchocerciasis: a randomized placebo-controlled study. Med Microbiol Immunol.

[10] Supali T, Djuardi Y, Pfarr KM, Wibowo H, Taylor MJ, Hoerauf A, Houwing-Duistermaat JJ, Yazdanbakhsh M, Sartono E (2008). Doxycycline treatment of *Brugia malayi*-infected persons reduces microfilaremia and adverse reactions after diethylcarbamazine and albendazole treatment. Clin Infect Dis.

[11] Foster J, Ganatra M, Kamal I, Ware J, Makarova K, Ivanova N, Bhattacharyya A, Kapatral V, Kumar S, Posfai J (2005). The *Wolbachia* genome of *Brugia malayi*: endosymbiont evolution within a human pathogenic nematode. PLoS Biol.

[12] Dunning Hotopp JC, Lin M, Madupu R, Crabtree J, Angiuoli SV, Eisen JA, Seshadri R, Ren Q, Wu M, Utterback TR (2006). Comparative genomics of emerging human ehrlichiosis agents. PLoS Genet.

[13] Brownlie JC, Adamski M, Slatko B, McGraw EA (2007). Diversifying selection and host adaptation in two endosymbiont genomes. BMC Evol Biol.

[14] Godel C, Kumar S, Koutsovoulos G, Ludin P, Nilsson D, Comandatore F, Wrobel N, Thompson M, Schmid CD, Goto S (2012). The genome of the heartworm, *Dirofilaria immitis*, reveals drug and vaccine targets. Faseb J.

[15] Choi YJ, Ghedin E, Berriman M, McQuillan J, Holroyd N, Mayhew GF, Christensen BM, Michalski ML (2011). A deep sequencing approach to comparatively analyze the transcriptome of lifecycle stages of the filarial worm, *Brugia malayi*. PLoS Negl Trop Dis.

[16] Luck AN, Evans CC, Riggs MD, Foster JM, Moorhead AR, Slatko BE, Michalski ML (2014). Concurrent transcriptional profiling of *Dirofilaria immitis* and its *Wolbachia* endosymbiont throughout the nematode life cycle reveals coordinated gene expression. BMC Genomics.

[17] Rosa BA, Jasmer DP, Mitreva M (2014). Genome-wide tissue-specific gene expression, co-expression and regulation of co-expressed genes in adult nematode *Ascaris suum*. PLoS Negl Trop Dis.

[18] Giardine B, Riemer C, Hardison RC, Burhans R, Elnitski L, Shah P, Zhang Y, Blankenberg D, Albert I, Taylor J (2005). Galaxy: a platform for interactive large-scale genome analysis. Genome Res.

[19] Blankenberg D, Von Kuster G, Coraor N, Ananda G, Lazarus R, Mangan M, Nekrutenko A, Taylor J. Galaxy: a web-based genome analysis tool for experimentalists. Curr Protoc Mol Biol.*.*2010;4:19:Unit 19.10.11–21.doi.10.1002/0471142727.mb1910s89 10.1002/0471142727.mb1910s89 PMC4264107 20069535

[20] Goecks J, Nekrutenko A, Taylor J (2010). Galaxy: a comprehensive approach for supporting accessible, reproducible, and transparent computational research in the life sciences. Genome Biol.

[21] Andrews S. FastQC: a quality control tool for high throughput sequence data. 2010. http://www.bioinformatics.babraham.ac.uk/projects/fastqc.

[22] Trapnell C, Williams BA, Pertea G, Mortazavi A, Kwan G, van Baren MJ, Salzberg SL, Wold BJ, Pachter L (2010). Transcript assembly and quantification by RNA-Seq reveals unannotated transcripts and isoform switching during cell differentiation. Nat Biotechnol.

[23] Trapnell C, Pachter L, Salzberg SL (2009). TopHat: discovering splice junctions with RNA-Seq. Bioinformatics.

[24] Langmead B, Trapnell C, Pop M, Salzberg SL (2009). Ultrafast and memory-efficient alignment of short DNA sequences to the human genome. Genome Biol.

[25] Eisen MB, Spellman PT, Brown PO, Botstein D (1998). Cluster analysis and display of genome-wide expression patterns. Proc Natl Acad Sci U S A.

[26] Zheng Q, Wang XJ (2008). GOEAST: a web-based software toolkit for Gene Ontology enrichment analysis. Nucleic Acids Res.

[27] Schleicher TR, VerBerkmoes NC, Shah M, Nyholm SV (2014). Colonization state influences the hemocyte proteome in a beneficial squid-Vibrio symbiosis. Mol Cell Proteomics.

[28] Darby C, Cosma CL, Thomas JH, Manoil C (1999). Lethal paralysis of *Caenorhabditis elegans* by *Pseudomonas aeruginosa*. Proc Natl Acad Sci U S A.

[29] Waterston RH, Thomson JN, Brenner S (1980). Mutants with altered muscle structure of *Caenorhabditis elegans*. Dev Biol.

[30] Moerman DG, Benian GM, Barstead RJ, Schriefer LA, Waterston RH (1988). Identification and intracellular localization of the unc-22 gene product of *Caenorhabditis elegans*. Genes Dev.

[31] Vogel BE, Hedgecock EM (2001). Hemicentin, a conserved extracellular member of the immunoglobulin superfamily, organizes epithelial and other cell attachments into oriented line-shaped junctions. Development.

[32] Vogel BE, Muriel JM, Dong C, Xu X (2006). Hemicentins: what have we learned from worms?. Cell Res.

[33] Avery L (1993). The genetics of feeding in *Caenorhabditis elegans*. Genetics.

[34] Raizen DM, Lee RY, Avery L (1995). Interacting genes required for pharyngeal excitation by motor neuron MC in *Caenorhabditis elegans*. Genetics.

[35] Van Gilst MR, Hadjivassiliou H, Jolly A, Yamamoto KR (2005). Nuclear hormone receptor NHR-49 controls fat consumption and fatty acid composition in *C. elegans*. PLoS Biol.

[36] Van Gilst MR, Hadjivassiliou H, Yamamoto KR (2005). A *Caenorhabditis elegans* nutrient response system partially dependent on nuclear receptor NHR-49. Proc Natl Acad Sci U S A.

[37] Hahn-Windgassen A, Van Gilst MR (2009). The *Caenorhabditis elegans* HNF4alpha Homolog, NHR-31, mediates excretory tube growth and function through coordinate regulation of the vacuolar ATPase. PLoS Genet.

[38] Coleman DL, Eicher EM (1990). Fat (fat) and tubby (tub): two autosomal recessive mutations causing obesity syndromes in the mouse. J Hered.

[39] Mukhopadhyay A, Deplancke B, Walhout AJ, Tissenbaum HA (2005). *C. elegans* tubby regulates life span and fat storage by two independent mechanisms. Cell Metab.

[40] Huang CC, Hall DH, Hedgecock EM, Kao G, Karantza V, Vogel BE, Hutter H, Chisholm AD, Yurchenco PD, Wadsworth WG (2003). Laminin alpha subunits and their role in *C. elegans* development. Development.

[41] Darby AC, Armstrong SD, Bah GS, Kaur G, Hughes MA, Kay SM, Koldkjaer P, Rainbow L, Radford AD, Blaxter ML (2012). Analysis of gene expression from the *Wolbachia* genome of a filarial nematode supports both metabolic and defensive roles within the symbiosis. Genome Res.

[42] Armstrong SD, Babayan SA, Lhermitte-Vallarino N, Gray N, Xia D, Martin C, Kumar S, Taylor DW, Blaxter ML, Wastling JM (2014). Comparative Analysis of the Secretome from a Model Filarial Nematode (*Litomosoides sigmodontis*) Reveals Maximal Diversity in Gravid Female Parasites. Mol Cell Proteomics.

[43] Prusse A, Vollmer S, Diesfeld HJ (1983). Immunocytochemical and ultrastructural studies on *Dipetalonema viteae* (Filarioidea). J Helminthol.

[44] Kostrouchova M, Krause M, Kostrouch Z, Rall JE (1998). CHR3: a *Caenorhabditis elegans* orphan nuclear hormone receptor required for proper epidermal development and molting. Development.

[45] Kouns NA, Nakielna J, Behensky F, Krause MW, Kostrouch Z, Kostrouchova M (2011). NHR-23 dependent collagen and hedgehog-related genes required for molting. Biochem Biophys Res Commun.

[46] Kostrouchova M, Krause M, Kostrouch Z, Rall JE (2001). Nuclear hormone receptor CHR3 is a critical regulator of all four larval molts of the nematode *Caenorhabditis elegans*. Proc Natl Acad Sci U S A.

[47] Starich TA, Herman RK, Shaw JE (1993). Molecular and genetic analysis of unc-7, a *Caenorhabditis elegans* gene required for coordinated locomotion. Genetics.

[48] Barnes TM, Hekimi S (1997). The *Caenorhabditis elegans* avermectin resistance and anesthetic response gene unc-9 encodes a member of a protein family implicated in electrical coupling of excitable cells. J Neurochem.

[49] Van Auken K, Weaver D, Robertson B, Sundaram M, Saldi T, Edgar L, Elling U, Lee M, Boese Q, Wood WB (2002). Roles of the Homothorax/Meis/Prep homolog UNC-62 and the Exd/Pbx homologs CEH-20 and CEH-40 in i embryogenesis. Development.

[50] Roayaie K, Crump JG, Sagasti A, Bargmann CI (1998). The G alpha protein ODR-3 mediates olfactory and nociceptive function and controls cilium morphogenesis in *C. elegans* olfactory neurons. Neuron.

[51] Crossgrove K, Maina CV, Robinson-Rechavi M, Lochner MC (2008). Orthologues of the *Drosophila melanogaster* E75 molting control gene in the filarial parasites *Brugia malayi* and *Dirofilaria immitis*. Mol Biochem Parasitol.

[52] Mimoto A, Fujii M, Usami M, Shimamura M, Hirabayashi N, Kaneko T, Sasagawa N, Ishiura S (2007). Identification of an estrogenic hormone receptor in *Caenorhabditis elegans*. Biochem Biophys Res Commun.

[53] Kwon JY, Kim-Ha J, Lee BJ, Lee J (2001). The MED-7 transcriptional mediator encoded by let-49 is required for gonad and germ cell development in *Caenorhabditis elegans*. FEBS Lett.

[54] Chen L, Ong B, Bennett V (2001). LAD-1, the *Caenorhabditis elegans* L1CAM homologue, participates in embryonic and gonadal morphogenesis and is a substrate for fibroblast growth factor receptor pathway-dependent phosphotyrosine-based signaling. J Cell Biol.

[55] Wen C, Levitan D, Li X (2000). Greenwald I: spr-2, a suppressor of the egg-laying defect caused by loss of sel-12 presenilin in *Caenorhabditis elegans*, is a member of the SET protein subfamily. Proc Natl Acad Sci U S A.

[56] Unhavaithaya Y, Shin TH, Miliaras N, Lee J, Oyama T, Mello CC (2002). MEP-1 and a homolog of the NURD complex component Mi-2 act together to maintain germline-soma distinctions in *C. elegans*. Cell.

[57] Karashima T, Sugimoto A, Yamamoto M (2000). *Caenorhabditis elegans* homologue of the human azoospermia factor DAZ is required for oogenesis but not for spermatogenesis. Development.

[58] Zhang Y, Foster JM, Nelson LS, Ma D, Carlow CK (2005). The chitin synthase genes chs-1 and chs-2 are essential for *C. elegans* development and responsible for chitin deposition in the eggshell and pharynx, respectively. Dev Biol.

[59] Choy RK, Thomas JH (1999). Fluoxetine-resistant mutants in *C. elegans* define a novel family of transmembrane proteins. Mol Cell.

[60] Heid PJ, Raich WB, Smith R, Mohler WA, Simokat K, Gendreau SB, Rothman JH, Hardin J (2001). The zinc finger protein DIE-1 is required for late events during epithelial cell rearrangement in *C. elegans*. Dev Biol.

[61] Tsuboi D, Qadota H, Kasuya K, Amano M, Kaibuchi K (2002). Isolation of the interacting molecules with GEX-3 by a novel functional screening. Biochem Biophys Res Commun.

[62] Kumar S, Chaudhary K, Foster JM, Novelli JF, Zhang Y, Wang S, Spiro D, Ghedin E, Carlow CK (2007). Mining predicted essential genes of *Brugia malayi* for nematode drug targets. PLoS One.

[63] Ellis RE, Kimble J (1995). The fog-3 gene and regulation of cell fate in the germ line of *Caenorhabditis elegans*. Genetics.

[64] Suh N, Jedamzik B, Eckmann CR, Wickens M, Kimble J (2006). The GLD-2 poly(A) polymerase activates gld-1 mRNA in the *Caenorhabditis elegans* germ line. Proc Natl Acad Sci U S A.

[65] L’Hernault SW, Shakes DC, Ward S (1988). Developmental genetics of chromosome I spermatogenesis-defective mutants in the nematode *Caenorhabditis elegans*. Genetics.

[66] Arduengo PM, Appleberry OK, Chuang P, L’Hernault SW (1998). The presenilin protein family member SPE-4 localizes to an ER/Golgi derived organelle and is required for proper cytoplasmic partitioning during *Caenorhabditis elegans* spermatogenesis. J Cell Sci.

[67] Singson A, Mercer KB, L’Hernault SW (1998). The *C. elegans* spe-9 gene encodes a sperm transmembrane protein that contains EGF-like repeats and is required for fertilization. Cell.

[68] Zugasti O, Rajan J, Kuwabara PE (2005). The function and expansion of the Patched- and Hedgehog-related homologs in *C. elegans*. Genome Res.

[69] Lim HH, Park BJ, Choi HS, Park CS, Eom SH, Ahnn J (1999). Identification and characterization of a putative *C. elegans* potassium channel gene (Ce-slo-2) distantly related to Ca(2+)-activated K(+) channels. Gene.

[70] Liu P, Ge Q, Chen B, Salkoff L, Kotlikoff MI, Wang ZW (2011). Genetic dissection of ion currents underlying all-or-none action potentials in *C. elegans* body-wall muscle cells. J Physiol.

[71] Smith WD (1993). Protection in lambs immunised with *Haemonchus contortus* gut membrane proteins. Res Vet Sci.

[72] Cachat E, Newlands GF, Ekoja SE, McAllister H, Smith WD (2010). Attempts to immunize sheep against *Haemonchus contortus* using a cocktail of recombinant proteases derived from the protective antigen, H-gal-GP. Parasite Immunol.

[73] McGonigle S, Yoho ER, James ER (2001). Immunisation of mice with fractions derived from the intestines of *Dirofilaria immitis*. Int J Parasitol.

[74] Lapierre LR, De Magalhaes Filho CD, McQuary PR, Chu CC, Visvikis O, Chang JT, Gelino S, Ong B, Davis AE, Irazoqui JE (2013). The TFEB orthologue HLH-30 regulates autophagy and modulates longevity in *Caenorhabditis elegans*. Nat Commun.

[75] Uno M, Honjoh S, Matsuda M, Hoshikawa H, Kishimoto S, Yamamoto T, Ebisuya M, Matsumoto K, Nishida E (2013). A fasting-responsive signaling pathway that extends life span in *C. elegans*. Cell Rep.

[76] Mango SE, Lambie EJ, Kimble J (1994). The pha-4 gene is required to generate the pharyngeal primordium of *Caenorhabditis elegans*. Development.

[77] Taubert S, Van Gilst MR, Hansen M, Yamamoto KR (2006). A Mediator subunit, MDT-15, integrates regulation of fatty acid metabolism by NHR-49-dependent and -independent pathways in *C. elegans*. Genes Dev.

[78] Watts JL, Browse J (1999). Isolation and characterization of a Delta 5-fatty acid desaturase from *Caenorhabditis elegans*. Arch Biochem Biophys.

[79] Picken NC, Eschenlauer S, Taylor P, Page AP, Walkinshaw MD (2002). Structural and biological characterisation of the gut-associated cyclophilin B isoforms from *Caenorhabditis elegans*. J Mol Biol.

[80] Joshua GW (2001). Functional analysis of leucine aminopeptidase in *Caenorhabditis elegans*. Mol Biochem Parasitol.

[81] Korolnek T, Zhang J, Beardsley S, Scheffer GL, Hamza I (2014). Control of metazoan heme homeostasis by a conserved multidrug resistance protein. Cell Metab.

[82] Davis MW, Fleischhauer R, Dent JA, Joho RH, Avery L (1999). A mutation in the *C. elegans* EXP-2 potassium channel that alters feeding behavior. Science.

[83] Mohrlen F, Hutter H, Zwilling R (2003). The astacin protein family in *Caenorhabditis elegans*. Eur J Biochem.

[84] Baylis HA, Furuichi T, Yoshikawa F, Mikoshiba K, Sattelle DB (1999). Inositol 1,4,5-trisphosphate receptors are strongly expressed in the nervous system, pharynx, intestine, gonad and excretory cell of *Caenorhabditis elegans* and are encoded by a single gene (itr-1). J Mol Biol.

[85] Walker DS, Gower NJ, Ly S, Bradley GL, Baylis HA (2002). Regulated disruption of inositol 1,4,5-trisphosphate signaling in *Caenorhabditis elegans* reveals new functions in feeding and embryogenesis. Mol Biol Cell.

[86] Han HF, Beckerle MC (2009). The ALP-Enigma protein ALP-1 functions in actin filament organization to promote muscle structural integrity in *Caenorhabditis elegans*. Mol Biol Cell.

[87] Goetinck S, Waterston RH (1994). The *Caenorhabditis elegans* muscle-affecting gene unc-87 encodes a novel thin filament-associated protein. J Cell Biol.

[88] Goetinck S, Waterston RH (1994). The *Caenorhabditis elegans* UNC-87 protein is essential for maintenance, but not assembly, of bodywall muscle. J Cell Biol.

[89] Kranewitter WJ, Ylanne J, Gimona M (2001). UNC-87 is an actin-bundling protein. J Biol Chem.

[90] Barstead RJ, Waterston RH (1989). The basal component of the nematode dense-body is vinculin. J Biol Chem.

[91] Ono K, Yu R, Mohri K, Ono S (2006). *Caenorhabditis elegans* kettin, a large immunoglobulin-like repeat protein, binds to filamentous actin and provides mechanical stability to the contractile apparatuses in body wall muscle. Mol Biol Cell.

[92] Forbes JG, Flaherty DB, Ma K, Qadota H, Benian GM, Wang K (2010). Extensive and modular intrinsically disordered segments in *C. elegans* TTN-1 and implications in filament binding, elasticity and oblique striation. J Mol Biol.

[93] Hiatt SM, Duren HM, Shyu YJ, Ellis RE, Hisamoto N, Matsumoto K, Kariya K, Kerppola TK, Hu CD (2009). *Caenorhabditis elegans* FOS-1 and JUN-1 regulate plc-1 expression in the spermatheca to control ovulation. Mol Biol Cell.

[94] Broday L, Kolotuev I, Didier C, Bhoumik A, Podbilewicz B, Ronai Z (2004). The LIM domain protein UNC-95 is required for the assembly of muscle attachment structures and is regulated by the RING finger protein RNF-5 in *C. elegans*. J Cell Biol.

[95] Bennuru S, Meng Z, Ribeiro JM, Semnani RT, Ghedin E, Chan K, Lucas DA, Veenstra TD, Nutman TB (2011). Stage-specific proteomic expression patterns of the human filarial parasite *Brugia malayi* and its endosymbiont *Wolbachia*. Proc Natl Acad Sci U S A.

[96] McKay SJ, Johnsen R, Khattra J, Asano J, Baillie DL, Chan S, Dube N, Fang L, Goszczynski B, Ha E (2003). Gene expression profiling of cells, tissues, and developmental stages of the nematode *C. elegans*. Cold Spring Harb Symp Quant Biol.

[97] Chen B, Liu P, Wang SJ, Ge Q, Zhan H, Mohler WA, Wang ZW (2010). alpha-Catulin CTN-1 is required for BK channel subcellular localization in *C. elegans* body-wall muscle cells. Embo J.

