# Supplementary figures and images for: Tissue-specific transcriptomics and proteomics of a filarial nematode and its Wolbachia endosymbiont
Source: BMC Genomics. 2015 Nov 11;16:920. doi: 10.1186/s12864-015-2083-2 (PMC4642636; doi:10.1186/s12864-015-2083-2)

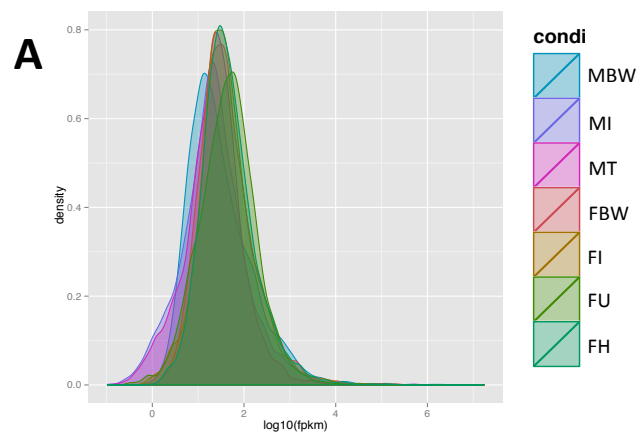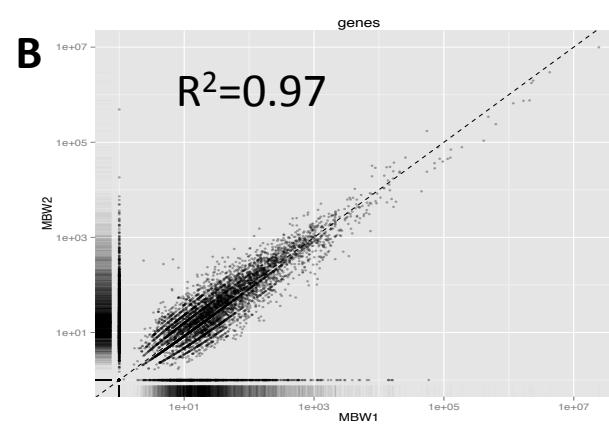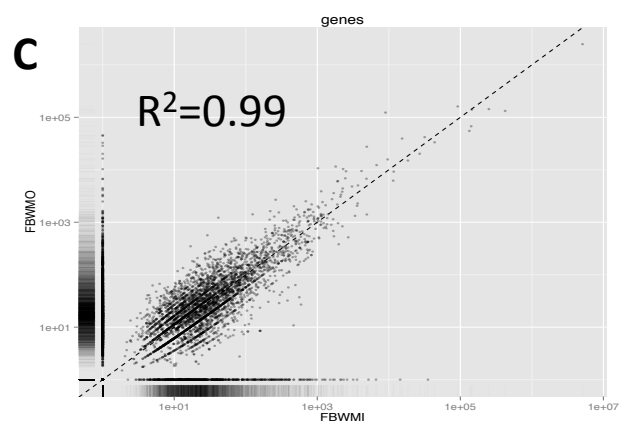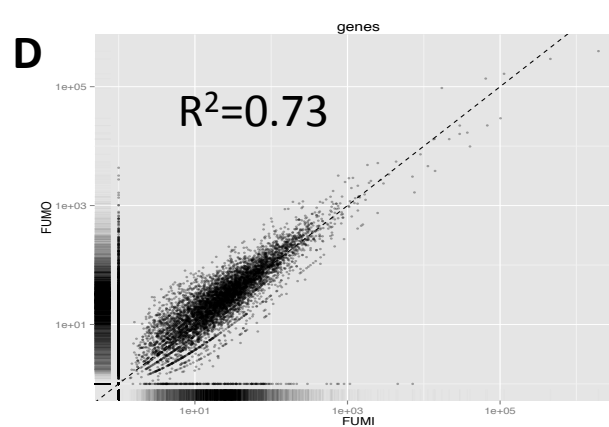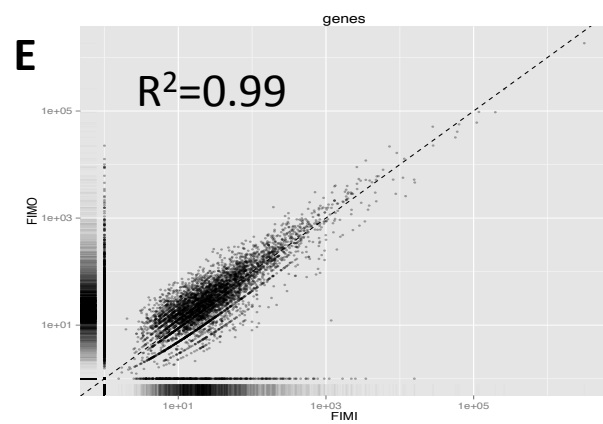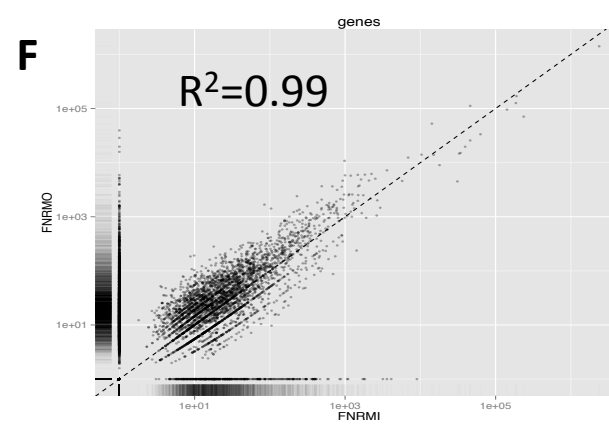

Supplement: Additional file 1: — Figure S1. Gene coverage and comparison of D. immitis biological tissue replicas. (PDF 25 kb) [file 12864_2015_2083_MOESM1_ESM.pdf]

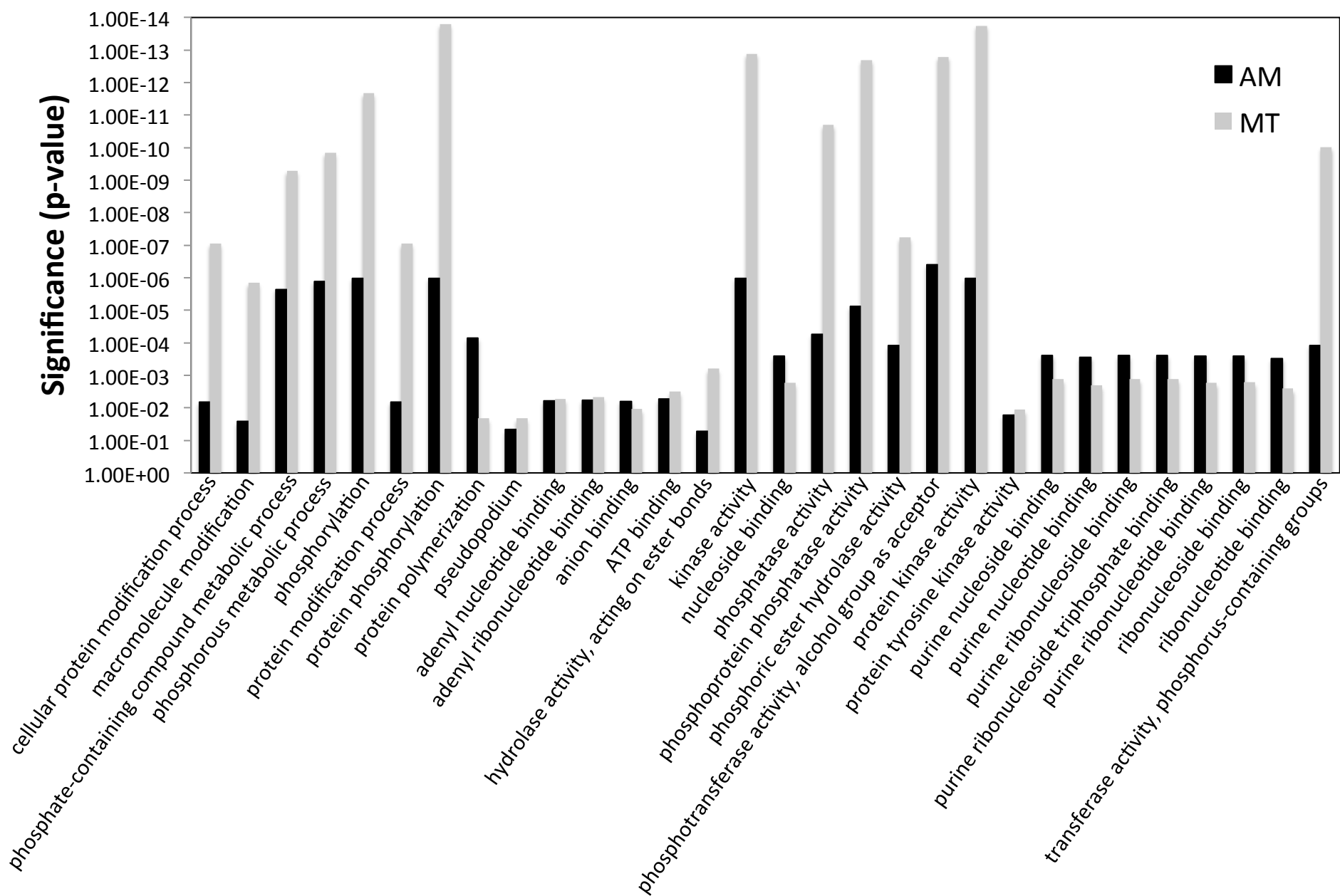

Supplement: Additional file 5: — Figure S2. Significance (p-value) of GO terms shared between D. immitis adult male and testestranscriptomes. (PDF 43 kb) [file 12864_2015_2083_MOESM5_ESM.pdf]

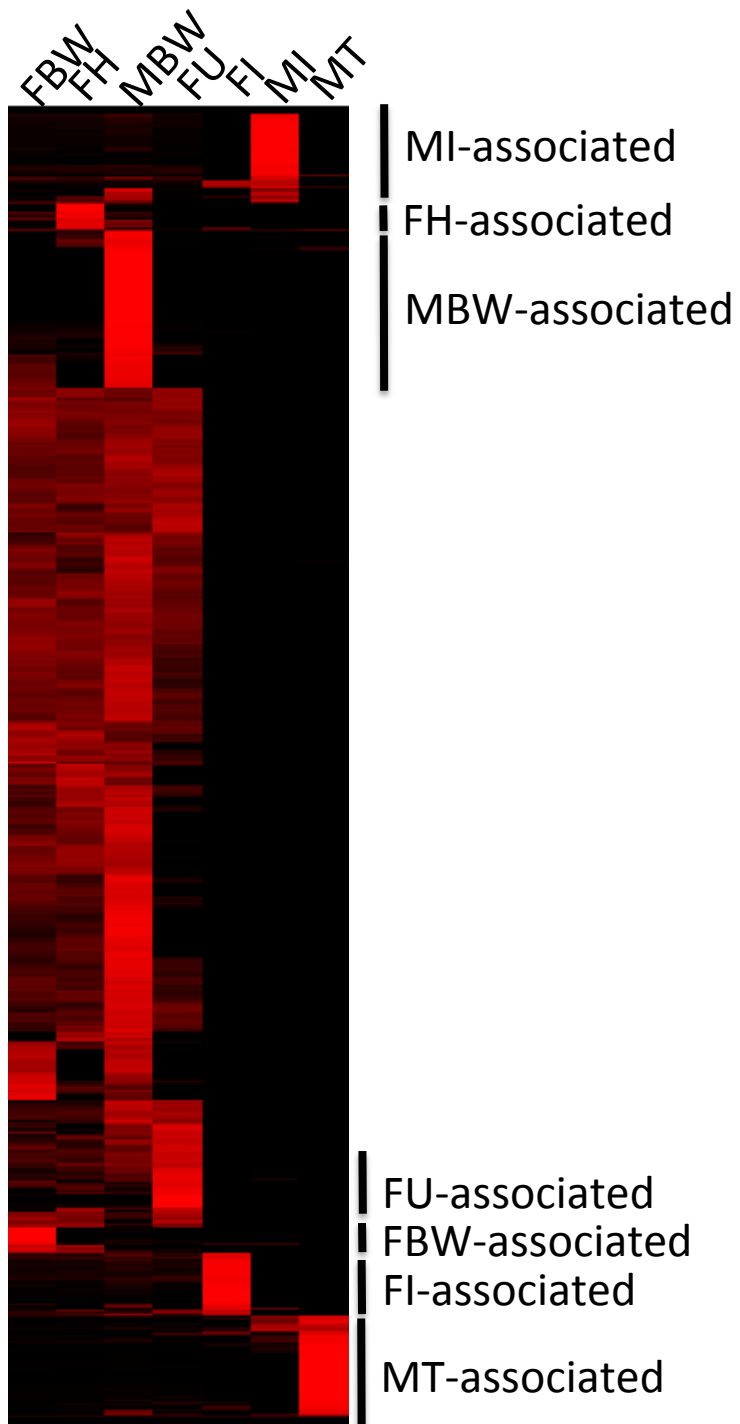

Supplement: Additional file 6: — Figure S3. Clustered transcriptomic data of Wolbachia gene expression profiles in D. immitis tissues. (PDF 21 kb) [file 12864_2015_2083_MOESM6_ESM.pdf]

FU FH FBW MH FI MS

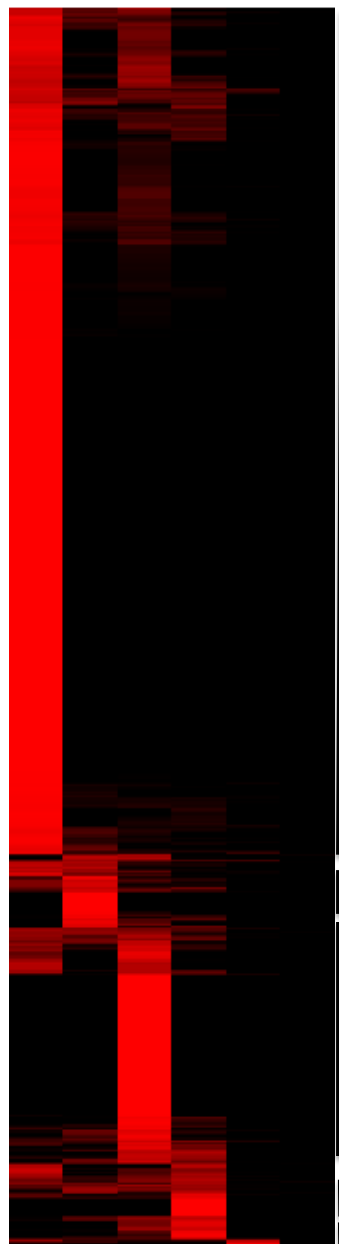

FU-associated

FH-associated

FBW-associated

MH-associated

FI-associated

Supplement: Additional file 13: — Figure S4. Clustered proteomic data (peptide spectral matches) of D. immitis proteins detected inD. immitis tissues. (PDF 50 kb) [file 12864_2015_2083_MOESM13_ESM.pdf]

**A**

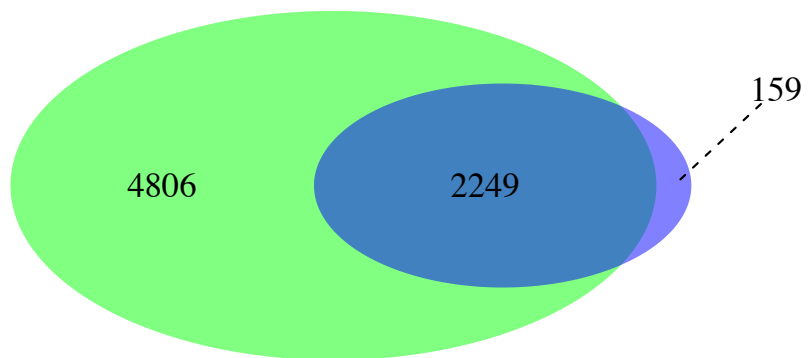

**B**

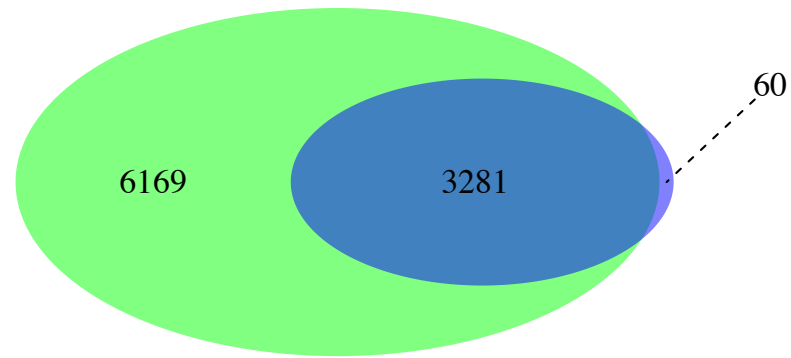

**C**

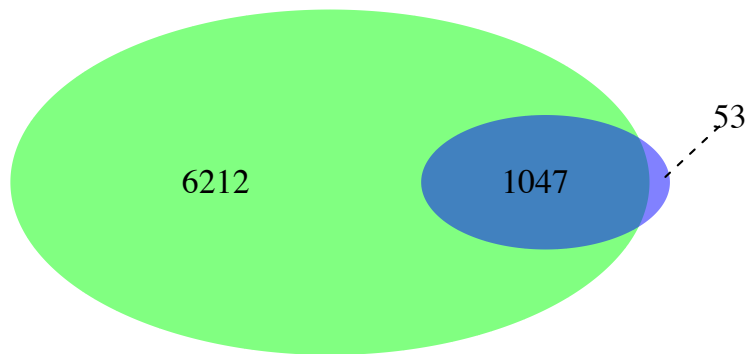

**D**

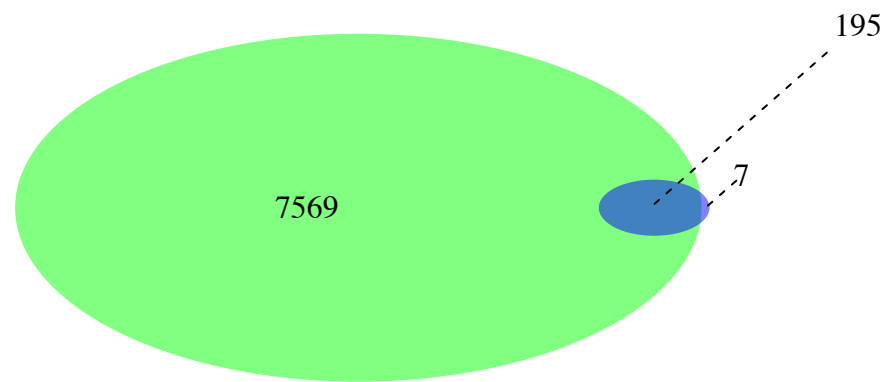

Supplement: Additional file 15: — Figure S5. Euler diagrams illustrating D. immitis genes detected by transcriptomic (green) and proteomic (blue) methods in various female D. immitis tissues: (A) body wall, (B) uterus, (C) head, (D) intestine. (PDF 1275 kb) [file 12864_2015_2083_MOESM15_ESM.pdf]

**A**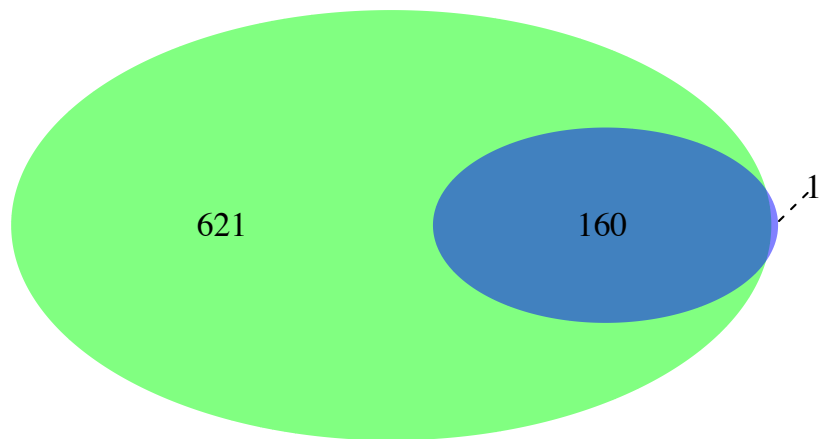**B**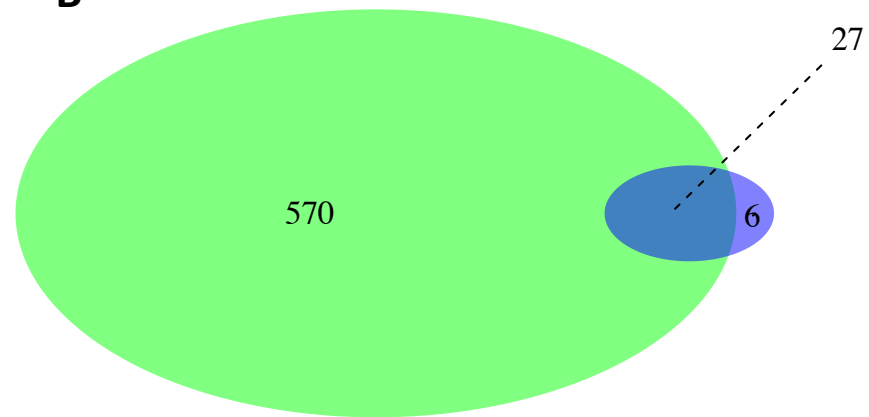**C**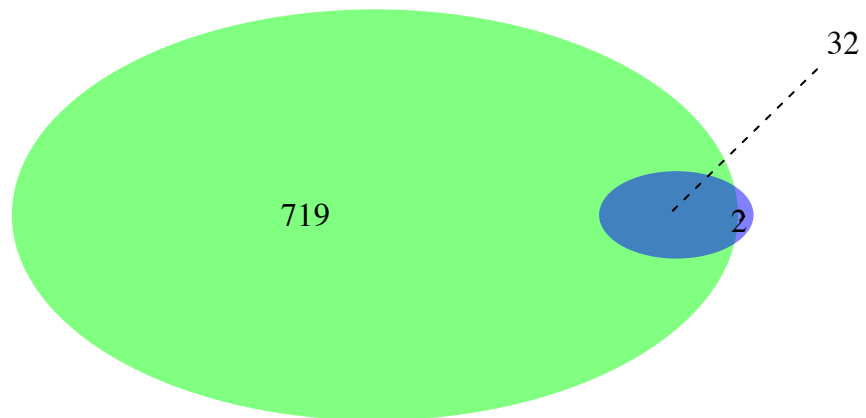

Supplement: Additional file 16: — Figure S6. Euler diagrams illustrating D. immitis genes detected by transcriptomic and proteomicmethods. (PDF 89 kb) [file 12864_2015_2083_MOESM16_ESM.pdf]
